# Novel Insights into the Influence of Seed Sarcotesta Photosynthesis on Accumulation of Seed Dry Matter and Oil Content in *Torreya grandis* cv. “Merrillii”

**DOI:** 10.3389/fpls.2017.02179

**Published:** 2018-01-09

**Authors:** Yuanyuan Hu, Yongling Zhang, Weiwu Yu, Heikki Hänninen, Lili Song, Xuhua Du, Rui Zhang, Jiasheng Wu

**Affiliations:** ^1^State Key Laboratory of Subtropical Silviculture, Zhejiang A & F University, Hangzhou, China; ^2^Key Laboratory of High Efficient Processing of Bamboo of Zhejiang Province, China National Bamboo Research Center, Hangzhou, China

**Keywords:** seed photosynthesis, sarcotesta, gas exchange, chlorophyll fluorescence, seed development, dry matter accumulation, oil content

## Abstract

Seed oil content is an important trait of nut seeds, and it is affected by the import of carbon from photosynthetic sources. Although green leaves are the main photosynthetic organs, seed sarcotesta photosynthesis also supplies assimilates to seed development. Understanding the relationship between seed photosynthesis and seed development has theoretical and practical significance in the cultivation of *Torreya grandis* cv. “Merrillii.” To assess the role of seed sarcotesta photosynthesis on the seed development, anatomical and physiological traits of sarcotesta were measured during two growing seasons in the field. Compared with the attached current-year leaves, the sarcotesta had higher gross photosynthetic rate at the first stage of seed development. At the late second stage of seed development, sarcotesta showed down-regulation of PSII activity, as indicated by significant decrease in the following chlorophyll fluorescence parameters: the maximum PSII efficiency (*F*_*v*_*/F*_*m*_), the PSII quantum yield (Φ_*PSII*_), and the photosynthetic quenching coefficient (*qP*). The ribulose 1, 5—bisphosphate carboxylase (Rubisco) activity, the total chlorophyll content (Chl_(a+b)_) and nitrogen content in the sarcotesta were also significantly decreased during that period. Treatment with DCMU [3-(3,4-dichlorophenyl)-1,1-dimethylurea] preventing seed photosynthesis decreased the seed dry weight and the oil content by 25.4 and 25.5%, respectively. We conclude that seed photosynthesis plays an important role in the dry matter accumulation at the first growth stage. Our results also suggest that down-regulation of seed photosynthesis is a plant response to re-balance the source-sink ratio at the second growth stage. These results suggest that seed photosynthesis is important for biomass accumulation and oil synthesis of the *Torreya* seeds. The results will facilitate achieving higher yields and oil contents in nut trees by selection for higher seed photosynthesis cultivars.

## Introduction

Fruit and seed growth and the formation of storage reserves in them require much carbon substrate and energy through respiration. The fruits and seeds and the surrounding pericarp/seed coat have developed mechanisms to refix some of the respired carbon (Blanke and Lenz, 1989). Besides the carbon originating from leaf photosynthesis, internal recycling of respiratory CO_2_ by fruits and seeds provides an important additional contribution to meet the carbon requirements of the seeds and fruits (Aschan and Pfanz, 2003).

It has been frequently shown that carbon re-fixation in reproductive organs makes in many plant species a significant photosynthetic contribution to meeting the carbon requirement of their own growth (Quebedeaux and Chollet, 1975; Whiley et al., 1992; King et al., 1998; Proietti et al., 1999; Furbank et al., 2004; Imai and Ogawa, 2009; Lytovchenko et al., 2011; Hu et al., 2012; Hua et al., 2012; Xu et al., 2016). Seed oil synthesis is dependent upon the supply of photosynthate from photosynthetic organs of the seeds (Baud and Lepiniec, 2010). It has been also reported that the photosynthesis taking place in green embryos, or in the external green non-foliar organs (e.g., pericarp, pod, silique, peel), plays an important role in the regulation of crop oil content (Singal et al., 1995; Asokanthan et al., 1997; Ruuska et al., 2004; Schwender et al., 2004; Goffman et al., 2005; Allen et al., 2009; Hua et al., 2012; Wang et al., 2016). Therefore, enormous research efforts have been devoted to studying the traits and mechanisms of photosynthesis in reproductive organs when discussing their growth and development.

Fruits maintain a relatively high gross photosynthetic rate at the early stage of development; the rate then progressively decreases until fruit maturity in evergreen species (Proietti et al., 1999; Hieke et al., 2002; Imai and Ogawa, 2009). It is known that similarly as the chloroplasts in leaves, the chloroplasts in the outer layer of fruits and seeds contain well-organized grana thylakoids and starch grains (Platt-Aloia and Thompson, 1981; Bondada and Oosterhuis, 2003; Li et al., 2006). The number of the granular thylakoids decreases and the size of the plastoglobules increases during fruit development (Prebeg et al., 2008). Some enzymes and pigments involved in photosynthesis have been found in the external green non-foliar organs of the reproductive organs, for example, in the silique wall of rapeseed (Hua et al., 2012), the pod of soybean (Quebedeaux and Chollet, 1975), in the ear and awns of wheat (Martinez et al., 2003; Li et al., 2004), and in the peel of tomato and apple (Xu et al., 1997; Chen and Cheng, 2007). The photosynthetic rate of fruits and seeds has been estimated by CO_2_ gas exchange measurements (Wullschleger and Oosterhuis, 1990; Hua et al., 2012; Xu et al., 2016) and by oxygen electrode measurements (Chen and Cheng, 2007; Hu et al., 2012; Hiratsuka et al., 2015). The contribution of fruits and seeds photosynthesis to the yield has been also extensively studied by estimating the carbon budget of CO_2_ gas exchange or O_2_ evolution measurements (Dejong and Walton, 1989; Hieke et al., 2002; Imai and Ogawa, 2009; Hu et al., 2012), by shading (Maydup et al., 2010; Hu et al., 2012) and by using photosynthetic inhibitors, such as 3-(3,4-dichlorophenyl)-1,1-dimethylurea (DCMU) or paraquat (Maydup et al., 2010; Hua et al., 2012).

Tree nuts are rich in monounsaturated (MUFA) and polyunsaturated fatty acids (PUFA), which are recommended as an important resource of healthy diet in human populations throughout the world (O'Neil et al., 2012). *Torreya grandis* cv. “Merrillii” (later also “*Torreya*,” or the “*Torreya* cultivar”), a tree species with significant economic value, has drupe-like fruits that provide an example of seedoil nuts, with an oil content of 55%; about which 80% are unsaturated fatty acids Wang and Xiu (2005). Yu et al. (1986) reported that active chloroplasts are observed in the fresh outer layer (sarcotesta) of seeds of *Torreya*. The sarcotesta remain green for a long time during seed development. However, little information is available on the underlying physiological and biochemical mechanisms of photosynthesis in *Torreya* seeds and the relative contribution of seed photosynthesis to seed dry matter accumulation and oil synthesis during the seed development.

The aim of the present study is to (1) examine the potential photosynthetic mechanisms underlying the variable photosynthetic rate at the different seed development stages, and (2) to evaluate the role of seed photosynthesis in dry matter accumulation and oil synthesis in the seeds. Our understanding of the photosynthetic physiology and of the role of seed photosynthesis at the different stages of seed development will help to provide a theoretical basis for achieving higher nut yields and higher oil content by effective utilization of potential photosynthesis of seed sarcotesta.

## Materials and methods

### Plant materials and growth conditions

Nine *Torreya grandis* cv. “Merrillii” grafting trees planted at Yuqian Town, Lin'an City, Zhejiang Province, China (30°10′ N, 119°22′ E) were used in this study. The trees were established by using 1 year old *Torreya grandis* cv. “Merrillii” scion on 2 year old *Torreya grandis* Fort. Ex Lindl. root stock in 2001, and they began bearing seed since 2009. The trees were spaced in a 5 m × 5 m orientation and they received 62.4 kg N ha^−1^, 48 kg P ha^−1^, and 43.2 kg K ha^−1^ in March; and 31.2 kg N ha^−1^, 24 kg P ha^−1^, and kg 21.6 kg K ha^−1^ after seed harvest. Throughout the study, the trees were maintained with standard fertilization, irrigation, and pest control practices recommended for the *Torreya* cultivar (Dai et al., 2008; Zhang et al., 2017).

**Figure 1:**
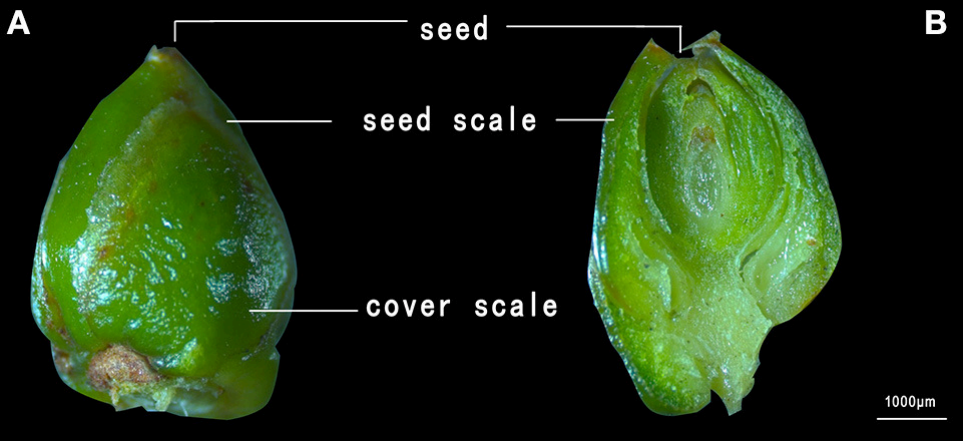
A microscopic photograph of a *Torreya grandis* cv. “Merrillii” seed before **(A)** and after **(B)** the protrusion of the seed from the seed scale.

The field measurements were carried out in two consecutive growing seasons during years 2015-2016. The development of the *Torreya* flowers and seeds lasted approximately 17 months, spanning 2 years. Accordingly, the seeds measured in 2015 and 2016 were based on blooming in 2014 and 2015, respectively. The blooming season lasted from middle April to early May both in 2014 and 2015. The seeds used in the study were produced by hand pollination on 12th April 2014 and 15th April 2015. The fertilization process is usually finished in early September (Liu et al., 2017). In present study, the viable seeds began to stick out from the seed scale on 15th April 2015 and 20th April 2016. Each of the seeds to be measured was labeled with a plastic tag on the day when the seed scale was breached and that day was documented as the day of protrusion for the seed (Figure 1). Seeds of approximately the same size were selected for the measurements.

### Design of the field study

With the exception of a separate experiment (see below), four *Torreya* trees were sampled for the measurements. From each sampled tree, seeds and leaves for the measurements were sampled randomly from the outer part of the crown at a height of approximately 1.0–1.5 m. The number of trees and seeds/leaves sampled are indicated below for each measurement category. Sampling took place on eight dates between 18th May and 9th September in 2015 and on six dates between 12th May and 11th September in 2016. However, some of the measurements were carried out only on the sampling dates in 2015 (see below), and sampling for the microscopic examination of the chloroplast ultrastructure was carried out only during one date in 2015. For each sampling date, the time of the measurement was determined as the number of days after the seeds emerged from the seed scale (days after seeds protrusion of, DASP).

A separate experiment with a photosynthesis inhibitor (DCMU,3-(3,4-dichlorophenyl)-1,1-dimethylurea) was carried out in 2015 with five *Torreya* trees not used in the other measurements (for details, see below).

### Measurements of the dimensions and weight of the seeds

For measurements of the dimensions and weight, three seeds from each of the four sampled trees were sampled on each sampling date in 2015 and 2016. Length (L), width (W) and thickness (T) of the seeds were measured with electronic digital calipers. Making the simplifying assumption that the seed shape is spherical, the surface area of the seeds was calculated by Equation (1) (Baryeh, 2001):

(1) $$\begin{array}{r} \text{S }=\;\pi \times {\left(\text{L }\times \text{ W }\times \text{ T}\right)}^{2/3} \end{array}$$

The seeds were weighed by an electronic balance. The seed fresh weight was determined immediately after sampling. After that, the seeds were dried at 80°C for more than 24 h until a constant weight (= dry weight) was attained. In order to examine qualitatively the color of the seeds, they were also photographed at each sampling occasion in 2015.

### Measurement of chlorophyll fluorescence in seeds

For measurements of chlorophyll fluorescence, three seeds from each of the four sampled trees were sampled in 2015 on four sampling dates at 27, 91, 106, 138, and 149 DASP. Chlorophyll fluorescence was measured in detached seeds with a pulse-modulated fluorometer PAM 2500 (Walz, Effeltrich, Germany). Seeds were wrapped in a wet paper towel after collecting them from the trees. We made the measurements in the middle part of the seeds where the surface is flat. Care was also taken to keep the distance between the fiber probe and the seed surface constant for all measurements. Prior to measurement, seeds were dark-adapted for 30 min. The minimal fluorescence (F_o_) was recorded after dark adaptation and the maximum fluorescence (F_m_) after a strong saturating pulse with the intensity and the width of 10,000 μmol m^−2^ s^−1^and 600 ms, respectively. Subsequently, each seed was illuminated with an actinic light of 1,161 μmol m^−2^s ^−1^ for 5 min, which allowed the fluorescent signal to reach a steady-state level (F_s_) before a saturating pulse was imposed to obtain the maximal fluorescence under light exposure (F_m_'). Shortly after the saturating pulse, the actinic light was turned off and a far-red light was switched on for 3 s to rapidly oxidize the primary quinone acceptor in PSII by drawing electrons from PSII to PSI, so that the minimal fluorescence (F_o_') under light exposure could be determined. The maximum PSII efficiency in dark-adapted seeds was calculated as F_v_/F_m_ = (F_m_ – F_o_)/F_m_ (Kitajima and Butler, 1975). The PSII quantum yield (Φ_*PSII*_) in the light was calculated as (F_m_' – F_s_)/F_m_' (Genty et al., 1989), and the photochemical quenching coefficient (*qP*) as (F_m_' – F_s_)/(F_m_' – F_o_') (Krause and Weis, 1991). Photochemical quantum yield of open PSII centers in the light-adapted state (F_v_'/F_m_') was calculated as (F_m_' – F_o_')/F_m_' (Schreiber et al., 1994).

### Measurement of leaf and seed CO_2_ exchange

For measurements of CO_2_ exchange, three seeds and their associated leaves from each of the four sampled trees were sampled on each sampling date. CO_2_ exchange was measured in the leaves and seeds using a portable photosynthesis measuring instrument (LI6400, LI–COR, Lincoln, NE, USA) according to Hu et al. (2013). Both for leaves and seeds, a cluster leaf chamber (6400-22, LI–COR) with an 18-RGB light source (LI–COR, Lincoln) was used.

In order to examine closely the seed photosynthetic parameters, seed CO_2_ exchange was measured at several PAR levels on all sampling dates in 2015. Subsequently, equations were fitted to the data by estimating the values of their coefficients representing the basic photosynthetic parameters.

Light response curves of seeds were determined as follows: seeds enclosed in the chamber were first kept at 1,200 μmol photon m^−2^ s^−1^ for at least 20 min; thereafter, PAR was decreased in a stepwise manner to measure the net photosynthetic rate (*P*_*n*_) at ten levels of photosynthetically active radiation (PAR): 0, 50, 100, 150, 200, 400, 600, 800, 1,000, and 1,200 μmol photons m^−2^ s^−1^. At each PAR level *P*_*n*_ was recorded when it reached a stable level, usually this took approximately 5 min. To avoid midday depression of photosynthesis in natural high-light (Larcher, 2003), the measurements were carried out in the field between 0800–1,100 and 1,400–1,600 h. The block temperature was maintained at 27–30°C for all measurements.

To calculate the *P*_(g,max)_ on a surface area, a dry weight or an individual seed basis, we used Equation (2) for calculating the net photosynthetic rate (*P*_*n*_) (Charles - Edwards, 1981):

(2) $$\begin{array}{r} P_{n}=\frac{b\text{PAR}}{1+\alpha \text{PAR}}-r \end{array}$$

Coefficient α is the reciprocal of PAR at which 50% of the asymptotic rate of *P*_*n*_ is attained; coefficient *b* is the gradient of the saturation curve of Equation (2) at the origin, that is, the maximum quantum yield. The dark respiration rate (*r*) was estimated as the CO_2_ exchange rate in darkness with PAR = 0 μmol photon m^−2^s^−1^. The gross photosynthetic rate (*P*_*g*_) of seeds was calculated by summing the net photosynthetic rate (*P*_n_) and the respiration rate (*r*). Finally, the maximum *P*_g_ at light saturation (*P*_(*g,max*)_) was calculated as *b*/α (Imai and Ogawa, 2009). For leaves, we used the standard method of expression *P*_*n*_ and *r* per one-sided leaf area, so that the leaf area was taken as one-half of the total two-sided surface area of the leaf. Therefore, for seeds *P*_g_ was divided by half of the surface area of the seed when *P*_g_ was expressed as per surface area. The *P*_(*g,max*)_ and *r*-values were calculated both on surface area (μmol CO_2_ m^−2^ s^−1^), seed dry weight (μmol CO_2_ g ^−1^ DW s^−1^), and individual seed (μmol CO_2_ seed ^−1^) basis. The apparent quantum use efficiency (AQE) was calculated by a linear regression on *P*_*g*_ on the PAR in the range PAR <200 μmol photon m^−2^s^−1^. The light saturation point (LSP) was determined using an AQ response curve analysis software (Version 1.0, LI-COR, 2/2008, Ranjan et al., 2012).

In order to compare seed and leaf gross photosynthesis at the same PAR, leaf gas exchange was measured at PAR = 800 μmol photons m^−2^ s^−1^ in 2015 and at PAR = 1,300 μmol photon m^−2^ s^−1^ in 2016. The net CO_2_ assimilation rates of leaves were recorded after equilibration to obtain the steady-state photosynthetic rate at the applied PAR level. In order to calculate the gross photosynthetic rate, the dark respiration rate of the leaves was also measured. For the seeds, we obtained the value of the net photosynthetic rate from their light response curves measured in 2015 at the same PAR level as used in the measurements with leaves (PAR = 800 μmol photons m^−2^s^−1^ and PAR = 1,300 μmol photons m^−2^s^−1^ in 2015 and 2016, respectively).

### Measurements of chlorophyll content, rubisco activity and nitrogen content of seed sarcotesta

For measurements of chlorophyll content, Rubisco (ribulose 1, 5 - bisphosphate carboxylase) activity, and nitrogen content, three seeds from each of four sampled trees were sampled on each sampling date (Rubisco and nitrogen content only in 2015). The sarcotesta (each about 25 mm^2^) were extracted with 5 ml 95% ethyl alcohol for 24 h in darkness. Absorbance of the supernatant was measured with a spectrophotometer (UV-2550, Shimadzu, Kyoto, Japan) at 649, 664, and 470 nm after centrifugation. The contents of total chlorophylls (Chl_a+b_), chlorophyll a (Chl_a_) and chlorophyll b (Chl_b_) were then calculated according to Lichtenthaler (1987).

The extraction of Rubisco was carried out according to Chen and Cheng (2003). Approximately 0.2 g of sarcotesta was ground with a liquid nitrogen pre-cooled mortar and pestled in 1.5 ml extraction buffer (50 mmol m^−3^ Hepes-KOH pH 10 mmol m^−3^ MgCl_2_, 2 mmol m^−3^ EDTA, 10 mmol m^−3^ dithiothreitol (DTT), 10% (v/v) Glycerol, 1% 1% (w/v) bovine serum albumin (BSA), and 1% Triton X-100). The extract was centrifuged at 13,000 × g for 5 min in an Eppendorf microcentrifuge at 4°C. An enzyme extract consisting of 100 mmol m^−3^ Bicine pH 8.0, 25 mmol m^−3^ KHCO_3_, 20 mmol m^−3^ MgCl_2_, 3.5 mmol m^−3^ ATP, 0.25 mmol m^−3^ NADH, 5 mmol m^−3^ phosphocreatine (PC), 17.5 Units per ml creatine phosphokinase (CPK), and 5 Units per ml both of Glyceraldehyde-3-phosphate-dehydrogenase (G3PD) and 3-phosphoglyceric phosphokinase (PGP) was immediately added to the reaction mixture to a final volume of 3 ml and incubated for 15 min at room temperature. The Rubisco activity was then measured at 340 nm using a spectrophotometer (UV-2550, Shimadzu, Kyoto, Japan). The reaction was initiated by addition of 0.25 mmol m^−3^ RuBP.

Sarcotesta samples were dried and ground, then the total nitrogen content was determined with an azotometer (Kjeltec-2300 FOSS, Sweden) according to the micro-Kjeldahl method (Schuman et al., 1972).

### Microscopy of the chloroplast ultrastructure in leaves and sarcotesta

Tissue samples (sarcotesta and leaf slices) were taken from seeds and the associated leaves at 81 DASP (5 July 2015) for the analysis of chloroplast ultrastructure. The samples were immediately fixed in 2.5% (v/v) glutaraldehyde (0.1 M phosphate buffer, pH 7.0) for at least 4 h once cut from the plants. The samples were then immersed in 1% (v/v) osmium tetroxide for post-fixation. The specimens were dehydrated using a graded series of ethanol and embedded in epoxy resin for ultrathin sectioning and transmission electron microscopy (H7650, HITACHI, Tokyo, Japan).

### Determining the effects of DCMU application on seed dry weight and seed quality

In order to further examine the role of seed photosynthesis in fulfilling the carbon requirements of the seeds, an experiment with DCMU [3-(3,4-dichlorophenyl)-1,1-dimethylurea] was carried out in 2015. DCMU is a chemical which is commonly used to inhibit Photosystem II in photosynthesis by binding to the D1 protein in the reaction center (Allen and Holmes, 1986; Chow et al., 1990). The seeds were treated with DCMU application according to Maydup et al. (2010) with some modifications. For this experiment, the labeled seeds (approximately 80 seeds from each tree) were divided into two parts. One part was treated with DCMU application, and the other part was used as a control so that it was treated otherwise similarly but without DCMU application. In detail, starting at 52 DASP (6th June), the experimental seeds were treated with 100 μmol m^−3^ of DCMU mixed with 0.1% (v/v) Tween-20 as a surfactant. Control seeds were treated with Tween-20 alone at the same concentration. The intact seed were immersed with DCMU solution in a fixed plastic valve bag for 30 min. During the immersing, the leaves were not allowed to be in contact with any DCMU solution. The DCMU application needed to decrease the photosynthetic electron transport rate (ETR) to approximately 30% was assessed by a pulse-modulated fluorometer PAM 2500 (Walz, Effeltrich, Germany) 1 day after application. Subsequently, the DCMU was applied at midday on sunny days (any rain may dilute the solution) once a week from 52 DASP until the seeds had reached maturity. The concentration of 100 μmol m^−3^ was chosen according to our preliminary experiment. Fan et al. (2007) also used this concentration in the solution with which spinach leaf discs were vacuum infiltrated with the aim of inhibiting Photosystem II and disrupting the redox poise needed for cyclic electron transport. Their study also shows that the DCMU application does not have any other effects beyond the inhibition of photosynthesis on plants. See supplementary Material as Table S1 for further details.

On 11th September 2015 the seeds were harvested and the length, the width, and the fresh weight (FW) of the intact seeds were measured immediately. Then the sarcotesta were removed from the seeds, and the fresh weight of the remaining hard seed was measured. After drying to a constant weight the dry weight (DW) of both the sarcotesta and the hard seed were measured. The sum of these two was taken as the dry weight of the intact seeds.

The relative contribution of the sarcotesta photosynthesis to the dry weight of the seeds was calculated as the dry weight difference between the control seeds and the seeds treated with DCMU, as a percentage of the dry weight of the control seeds. This was carried out separately both to the intact seeds and to the seeds with sarcotesta removed.

Finally, in order to measure the oil content, the kernel of both control and DCMU treated seeds was ground with liquid nitrogen. About 8 g (per extraction thimble made of thick filter paper) of full fat flour was defatted in a Soxhlet apparatus with petroleum ether solvent (boiling point range 38.7–54.8°C) for 8 h. The defatted samples were dried overnight (10–12 h) in a fume hood to remove residual petroleum ether and then weighed to calculate the oil content. The fatty acids composition of the oil was analyzed by gas chromatography (Agilent 7890A, Agilent Technologies, USA) according to the Fatty acid methyl esters (FAME) method of Maxwell and Marmer (1983). The standard gas chromatogram of main fatty acids is shown in Figure S1.

### Statistical analyses

Data were subjected to analysis of variance (ANOVA) using SPSS statistical software (16.0, IBM, New York, USA). The data are presented as the mean ± standard deviation (SD). The differences in the average values among different sampling occasions were tested by the least significant difference (LSD). Differences at *P* ≤ 0.05 were considered significant. The relationships between Rubisco activity, nitrogen content and *P*_(*g,max*)_ were determined using correlation analysis.

## Results

### Seed color, dimensions, and weight

**Figure 2:**
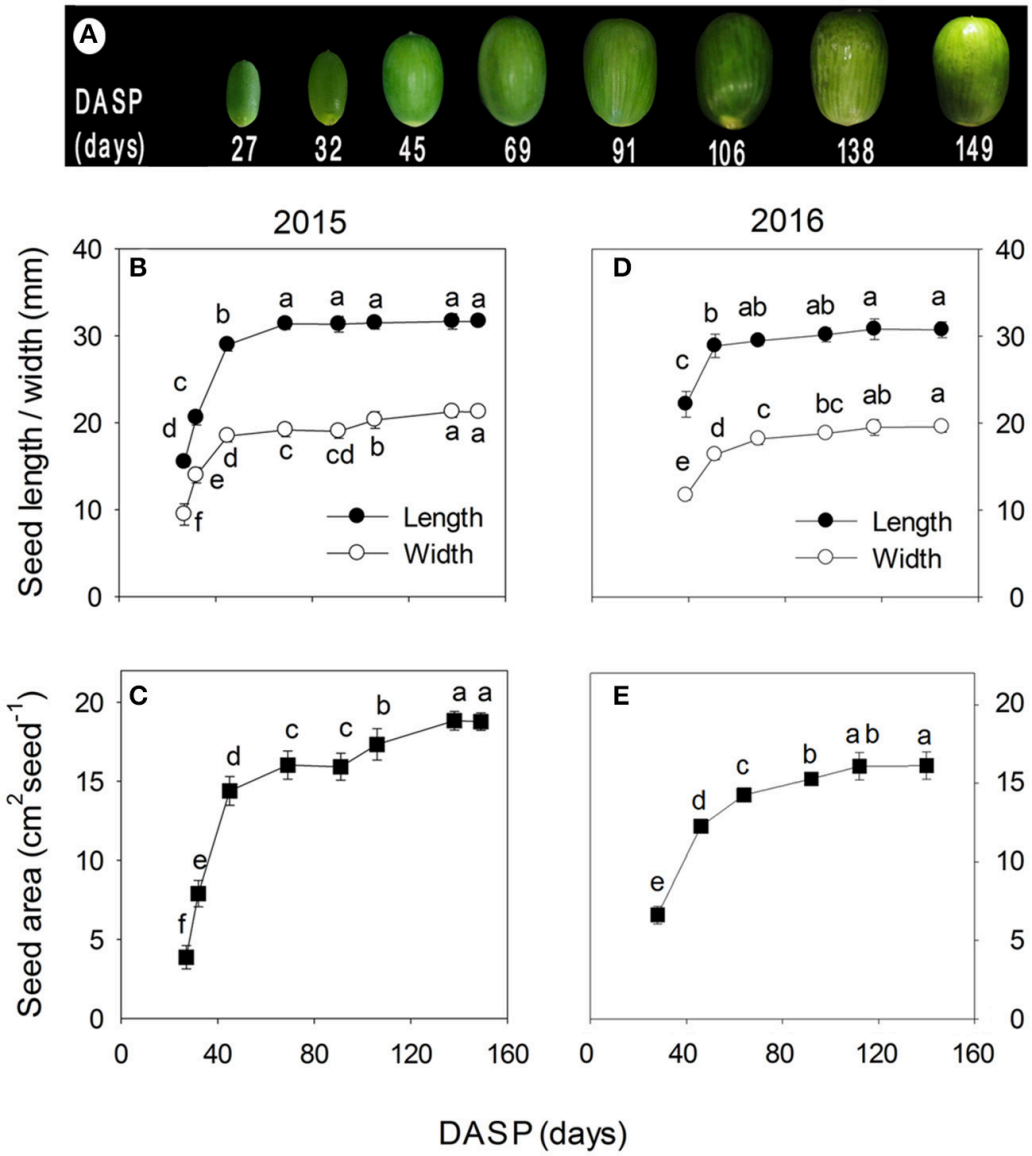
Seed development in *Torreya grandis* cv. “Merrillii” in 2015 and 2016, as indicated by the color **(A)**, the length and the width **(B,D)**, and the area **(C,E)** of the seeds. In **(B–E)** the mean ± SD is indicated. Different letters denote significant differences at *P* ≤ 0.05 level. *n* = 4 trees. DASP, days after seed protrusion.

The color of the seeds at 27 DASP was deep green, turned to yellowish green at the middle stage of seed development (about at 69 DASP), and then become again much greener with further seed development (Figure 2A). Similar patterns of the seed length, width, and area were found both in 2015 (Figures 2B,C) and 2016 (Figures 2D,E): They all showed first a somewhat linear increase at the early stage, but later the increase leveled off before the maximum values were attained.

**Figure 3:**
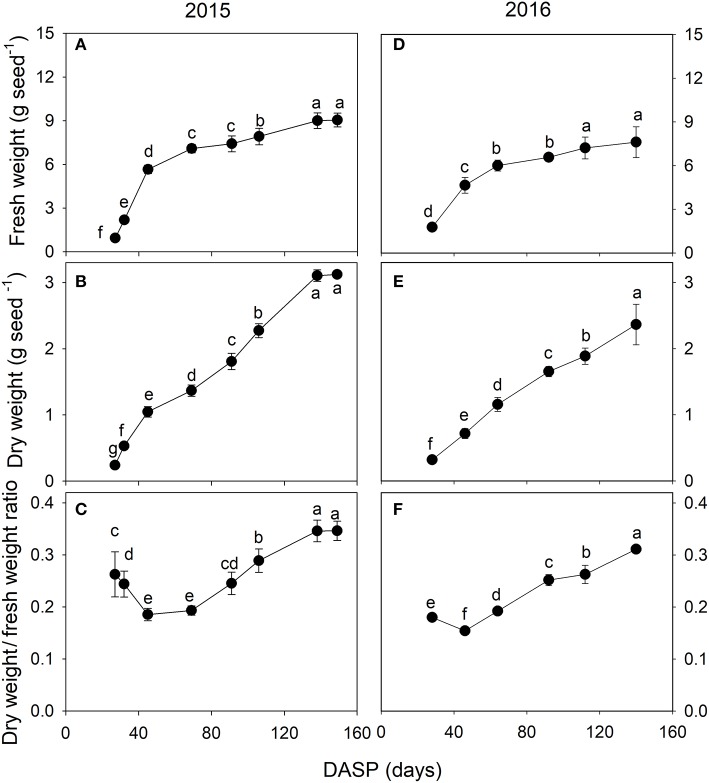
Fresh weight **(A,D)**, dry weight **(B,E)** and dry weight/fresh weight ratio **(C,F)** of seeds of *Torreya grandis* cv. “Merrillii” during seed development in 2015 and 2016. For each quantity the mean ± SD is indicated. Different letters denote significant differences at *P* ≤ 0.05 level. *n* = 4 trees. DASP, days after seed protrusion.

The seed fresh and dry weights exhibited a significant increasing trend during the seed development. The fresh weight of the seeds increased rapidly from 27 to 45 DASP in 2015 with a mean of 0.3 g day^−1^ (Figure 3A) and from 28 to 46 DASP in 2016 with a mean of 0.2 g day^−1^ (Figure 3D), followed in both years by a slower increase before reaching a constant weight. The seed dry weight significantly increased by 471 and 126% from 27 to 69 DASP and 70 to 149 DASP, respectively (Figure 3B). It also showed a more linear increasing trend from 28 to 140 DASP in 2016 (Figure 3E). The mean seed dry/fresh weight ratio was in 2015 the lowest at 45 DASP, followed first a plateau for approximately 24 days, then by an increase until 138 DASP, and remained finally another plateau thereafter (Figure 3C). Similarly, the mean seed dry/fresh weight ratio was the lowest at 46 DASP in 2016 (Figure 3F).

### CO_2_ exchange in the seeds

**Figure 4:**
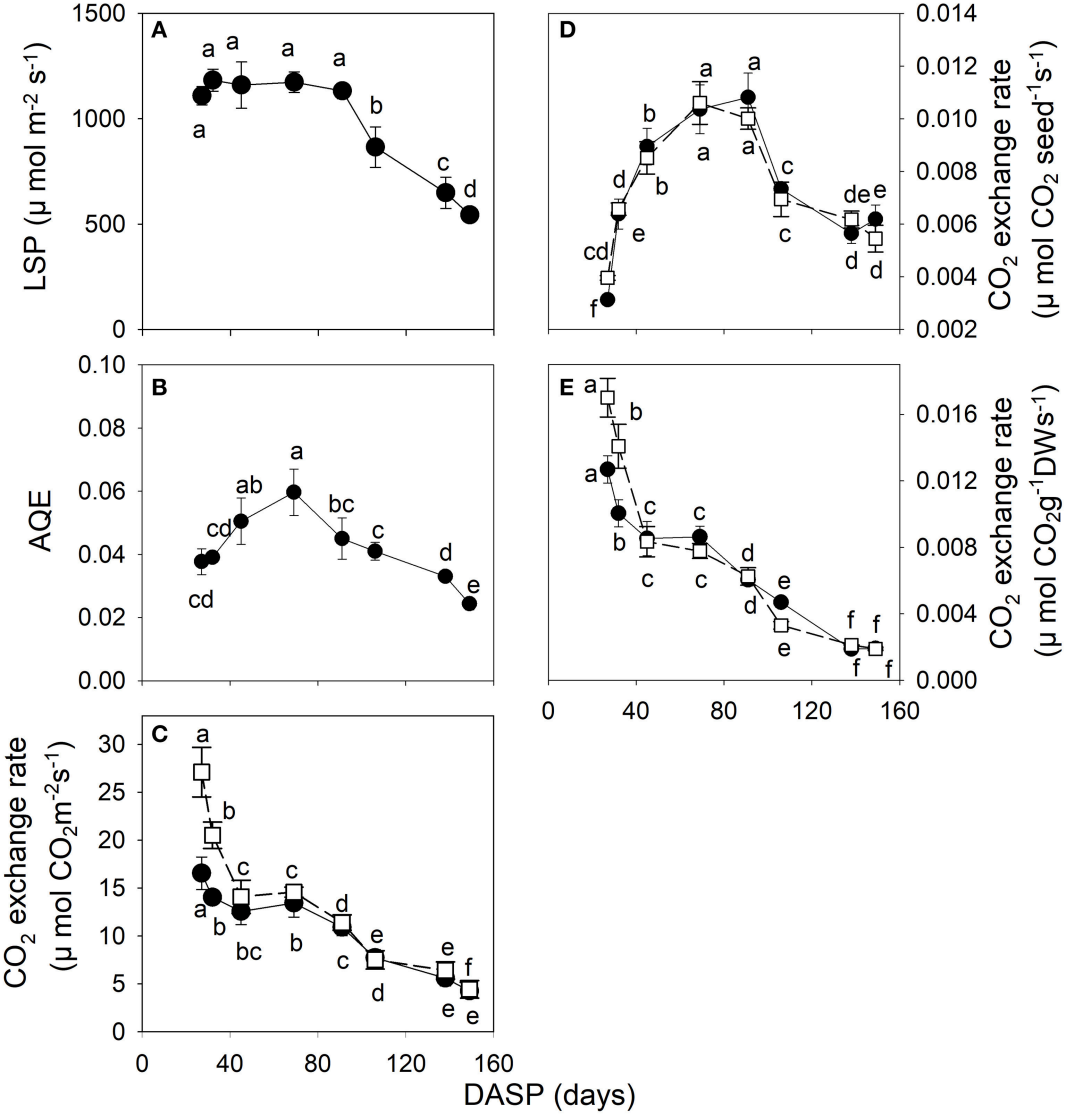
CO_2_ exchange parameters in seeds of *Torreya grandis* cv. “Merrillii” during seed development in 2015. The light saturation point LSP **(A)**, the apparent quantum use efficiency AQE **(B)**, the rate of CO_2_ exchange on surface area **(C)**, single seed **(D)** and dry weight **(E)** basis. In the rate of CO_2_ exchange **(C–E)**, both the maximal rate of gross photosynthesis at light saturation (*P*_(*g,max*)_, filled circles) and respiration rate (*R*_*d*_, open squares) are indicated. For each quantity the mean ± SD is indicated. Different letters denote significant differences at *P* ≤ 0.05 level. *n* = 4 trees. DASP, days after seed protrusion.

The photosynthetic light response curves of seeds during their developmental stages are shown in Supplementary Data Figure S2. From 27 to 91 DASP the seeds had a high light saturation point (LSP) of 1,110~1,183 μmol m^−2^ s^−1^. Then, the LSP significantly decreased with seed age greater than 91 DASP (Figure 4A). The apparent quantum use efficiency (AQE) of seeds showed an increasing trend from 27 to 69 DASP, followed by a decreasing trend as seed development progressed (Figure 4B). The dark respiration *R*_*d*_ and the maximum rate of gross photosynthesis *P*_(*g,max*)_ per seed surface area rapidly decreased from 27 to 45 DASP, then stabilized for about 24 days, followed by another decrease until seed-maturation (Figure 4C). The *R*_*d*_ and *P*_(*g,max*)_ per seed exhibited a dramatic increase from 27 to 91 DASP, followed by a pronounced decrease during the development of the seed (Figure 4D). The *R*_*d*_ and *P*_(*g,max*)_ per dry weight indicated a rapid decreasing trend from 27 to 45 DASP followed by a relatively stable value for about 24 days, and then another decrease as seed development proceeded (Figure 4E).

### Chlorophyll fluorescence in sarcotesta of the seeds

The maximum quantum yield of PSII (*F*_*v*_*/F*_*m*_) in the sarcotesta of the seeds was maintained at 0.74~0.76 from 27 to 138 DASP, after that the (*F*_*v*_*/F*_*m*_) significantly decreased with further seed development (Table 1). The actual photochemical efficiency of PSII (Φ_*PSII*_), the photochemical quenching coefficient (*qP*) and the photochemical quantum yield of open PSII centers in the light-adapted state (*F*_*v*_'*/F*_*m*_') maintained a relatively constant value from 27 to 91 DASP. However, the Φ_*PSII*_ and *qP* significantly decreased from 91 to 106 DASP, while the *F*_*v*_'*/F*_*m*_' was constant. The *F*_*v*_*/F*_*m*_in the sarcoteata of the seed was significantly lower at 138 DASP than that at 106 DASP. All of the parameters Φ_*PSII*_, *qP* and *F*_*v*_'*/F*_*m*_' significantly decreased from 138 to 149 DASP (Table 1).

**Table 1:** Chlorophyll fluorescence parameters of seed sarcotesta in *Torreya grandis* cv. “Merrillii” during seed development in 2015.

**DAPS (days)**	***F_*v*_/F_*m*_* (relative unit)**	***Φ_*PSII*_* (relative unit)**	***qP* (relative unit)**	***F_*v*_'/F_*m*_'* (relative unit)**
27	0.74 ± 0.004^ab^	0.24 ± 0.018^a^	0.36 ± 0.015^a^	0.60 ± 0.013^a^
91	0.75 ± 0.004^ab^	0.24 ± 0.012^a^	0.37 ± 0.014^a^	0.60 ± 0.002^a^
106	0.76 ± 0.014^a^	0.21 ± 0.016^b^	0.33 ± 0.033^b^	0.61 ± 0.025^a^
138	0.74 ± 0.006^b^	0.20 ± 0.015^b^	0.30 ± 0.017^b^	0.62 ± 0.023^a^
149	0.70 ± 0.006^c^	0.14 ± 0.002^c^	0.20 ± 0.014^c^	0.53 ± 0.010^c^

*DASP, days after seed protrusion*.

*Data are mean ± SD. Different letters denote significant differences at P ≤ 0.05 level. n = 4 trees. The Φ_PSII_, qP and F_v_'/F_m_' were measured at 1,126 μmol photon m^−2^s^−1^*.

### Chlorophyll content, rubisco activity and its relationship with *P*_(*g, max*)_ in the sarcotesta

**Figure 5:**
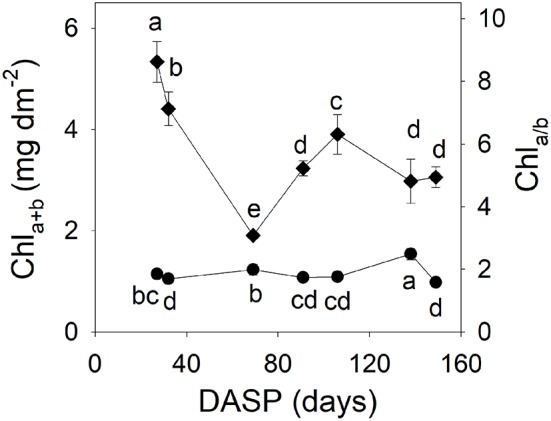
Total chlorophyll content Chl_a+b_ (left vertical axis, diamonds) and Chl_a/b_ ratio (right vertical axis, circles) in sarcotesta of seeds of *Torreya grandis* cv. “Merrillii” during seed development in 2015. Different letters denote significant differences at *P* ≤ 0.05 level. *n* = 4 trees. DASP, days after seed protrusion.

The total chlorophyll content (Chl_a+b_) per area of sarcotesta of the seeds showed a rapid decrease from 27 to 69 DAPS in 2015 (Figure 5). This initial drop was about 63% as compared with the first measured value (Figure 5). After that, the total chlorophyll content showed an increase from 70 to 106 DASP, and then a significant decrease as seed development proceeded. The Chl_a/b_ ratio of sarcotesta of the seeds remained relatively low for a long period, then reached its peak value at 138 DASP.

**Figure 6:**
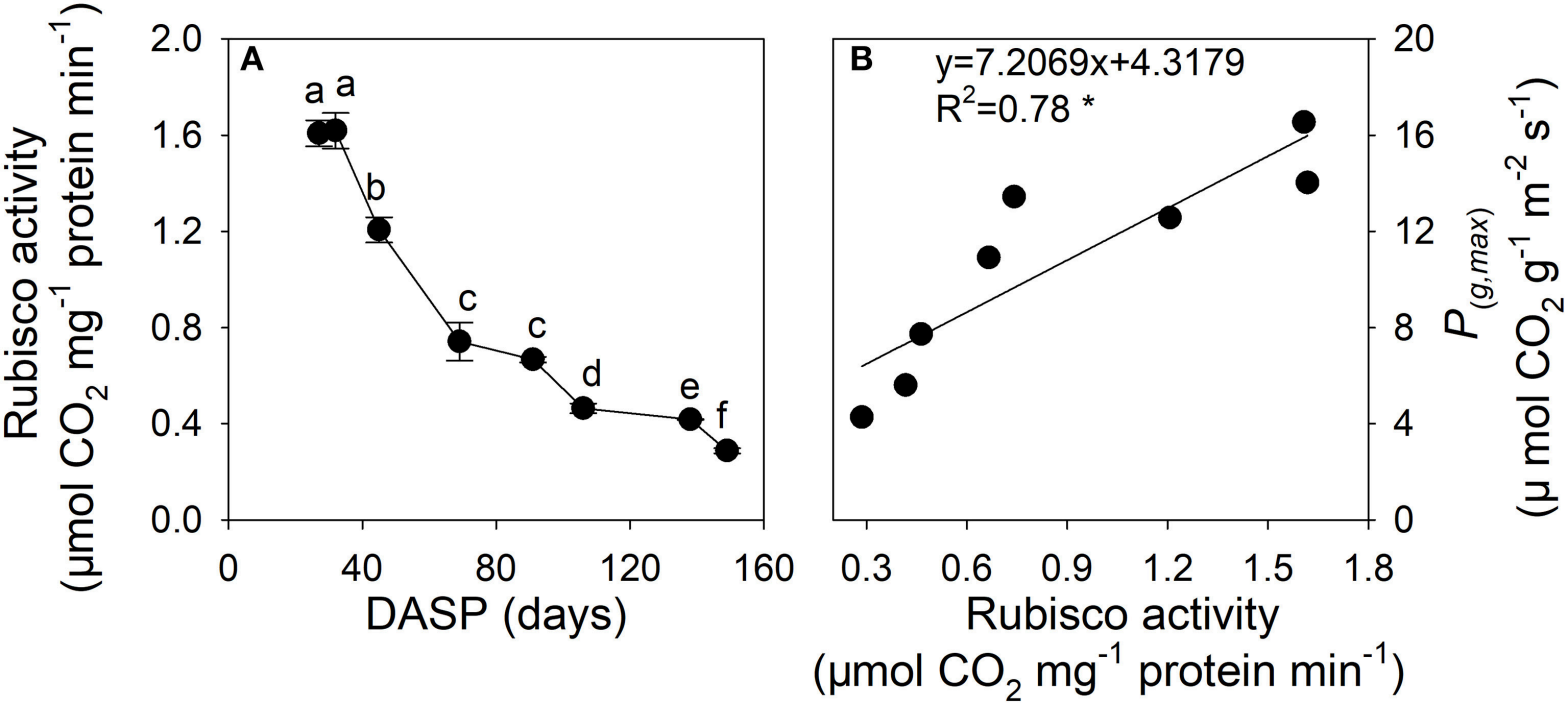
Ribulose-1,5-biphosphate carboxylase (Rubisco) activity **(A)** and the correlation between the maximal rate of gross photosynthesis at light saturation (*P*_(*g,max*)_) per area, and Rubisco activity **(B)** in sarcotesta of seeds of *Torreya grandis* cv. “Merrillii” during seed development in 2015. In **(A)** the mean ± SD is indicated. Different letters denote significant differences at *P* ≤ 0.05 level. *n* = 4 trees. DASP, days after seed protrusion. The solid line represent the best-fit linear regressions between the maximal rate of gross photosynthesis at light saturation (*P*_(*g,max*)_) per area, and Rubisco activity: ^*^P ≤ 0.05; ^**^P ≤ 0.01; ^***^ *P* ≤ 0.001; ns, not significant.

The Rubisco activity of sarcotesta of seed showed a decreasing trend during the seed development (Figure 6A). The reduction in Rubisco activity was 53.9% from 27 to 69 DASP and 43.8% from 70 to 138 DASP, respectively. The Rubisco activity of sarcotesta was significantly positively correlated with its *P*(_*g, max*_) (Figure 6B).

### Nitrogen (N) content in sarcotesta and its relationship with *P*_(*g, max*)_

**Figure 7:**
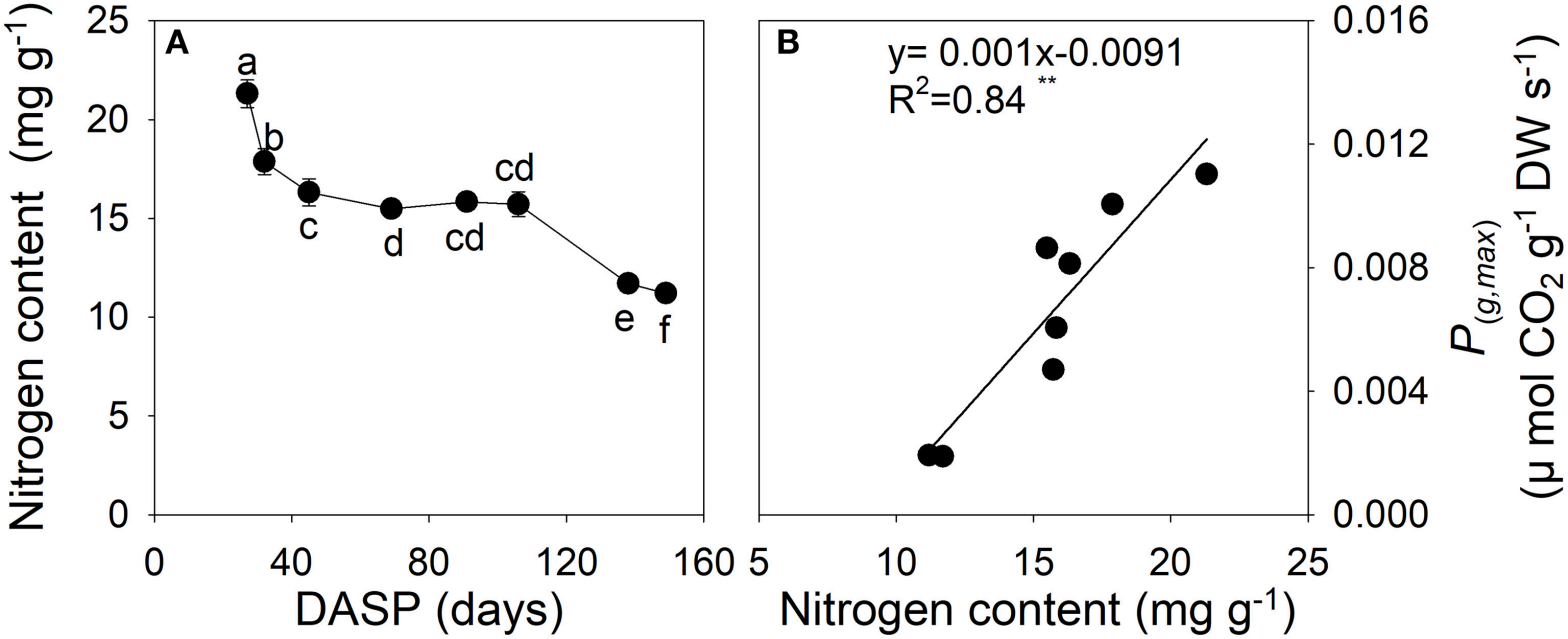
Nitrogen content **(A)** and the correlation between the maximal rate of gross photosynthesis at light saturation *P*_(*g,max*)_ per dry weight, and nitrogen content **(B)** in sarcotesta of seeds of *Torreya grandis* cv. “Merrillii” during seed development in 2015. In **(A)** the mean ± SD is indicated. Different letters denote significant differences at *P* ≤ 0.05 level. *n* = 4 trees. DASP, days after seed protrusion. The solid line represent the best-fit linear regressions between the maximal rate of gross photosynthesis at light saturation *P*_(*g,max*)_ per dry weight, and nitrogen content: ^*^P ≤ 0.05; ^**^P ≤ 0.01; ^***^P ≤ 0.001; ns, not significant.

The N content of sarcotesta showed a dramatic decrease from 27 to 69 DASP (Figure 7A). After that the N content level was constant until 106 DASP, after which it rapidly decreased as seed development proceeded (Figure 7A). The N content in sarcotesta was linearly and positively correlated with *P*_(*g,max*)_ per dry weight of the seeds (Figure 7B, *P* = 0.0013).

### Seed and leaf photosynthesis and the contribution of seed photosynthesis to seed carbon requirements

**Figure 8:**
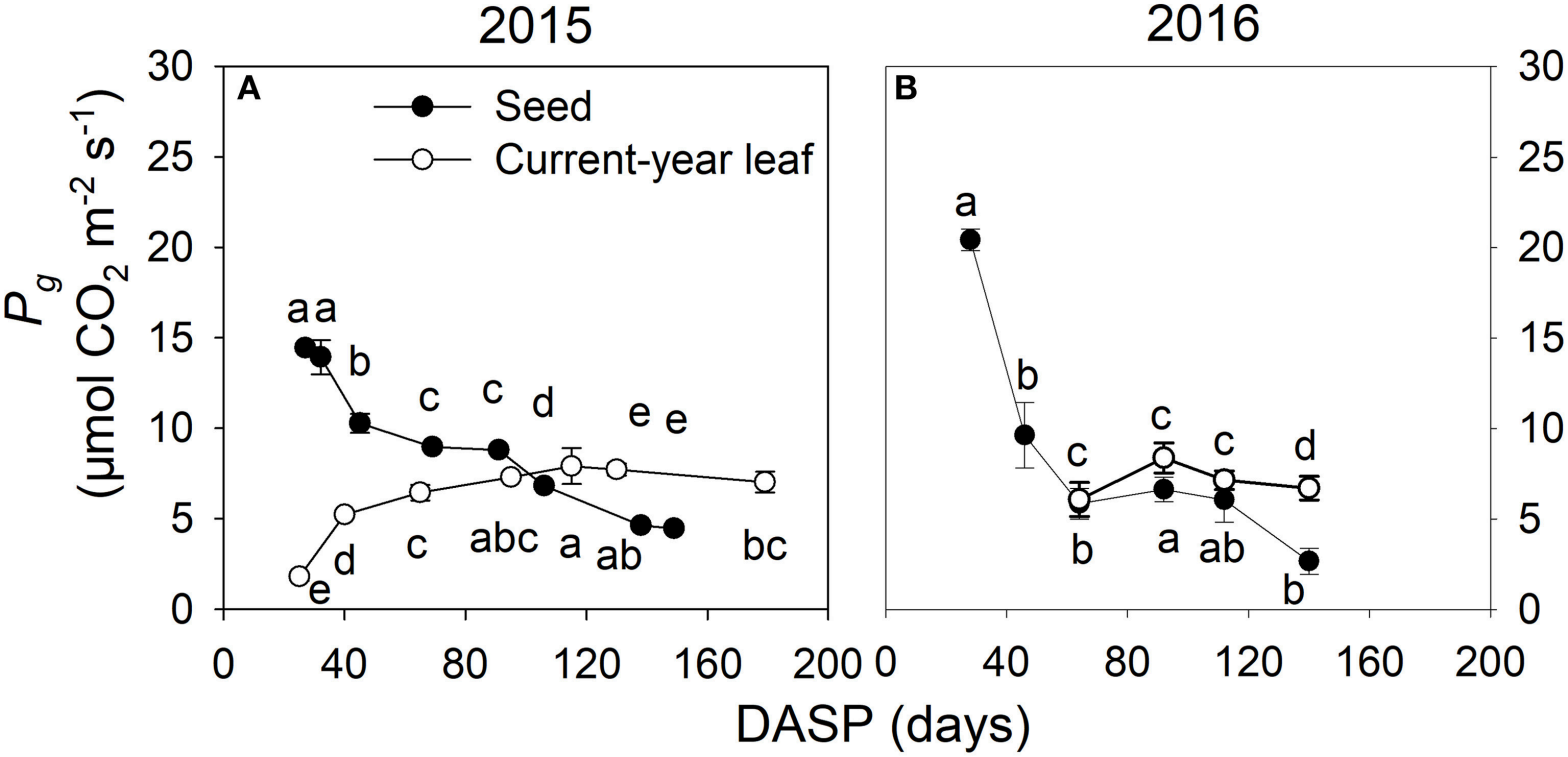
Gross photosynthetic rate (mean ± SD) in seeds (filled circles) and leaves (open circles) of *Torreya grandis* cv. “Merrillii” during growing season 2015 under PAR = 800 μmol m^−2^ s^−1^ **(A)** and during growing season 2016 under PAR= 1,300 μmol m^−2^ s^−1^ **(B)**. *n* = 4 trees. DASP, days after seed protrusion. Different letters denote significant differences in seeds during the seed development stages (up of the line and scatter plot), and in current-year leaves during the seed development stages (down of the line and scatter plot) at *P* ≤ 0.05 level.

Seed photosynthesis and leaf photosynthesis were compared by measuring both of them under the same PAR conditions. Similar patterns were found both in 2015 (Figure 8A) and 2016 (Figure 8B): In the seeds, the rate of gross photosynthesis *P*_*g*_ was very high at the beginning of the seed development. After that it decreased rapidly. In the leaves, a contrasting pattern from low rates of *P*_*g*_ at the beginning of the growing season to higher ones later was found. Accordingly, the rate of *P*_*g*_ was at early stages much higher in seeds than in leaves, whereas later this was reversed (Figure 8).

### Chloroplast ultrastructure in leaves and sarcotesta

**Figure 9:**
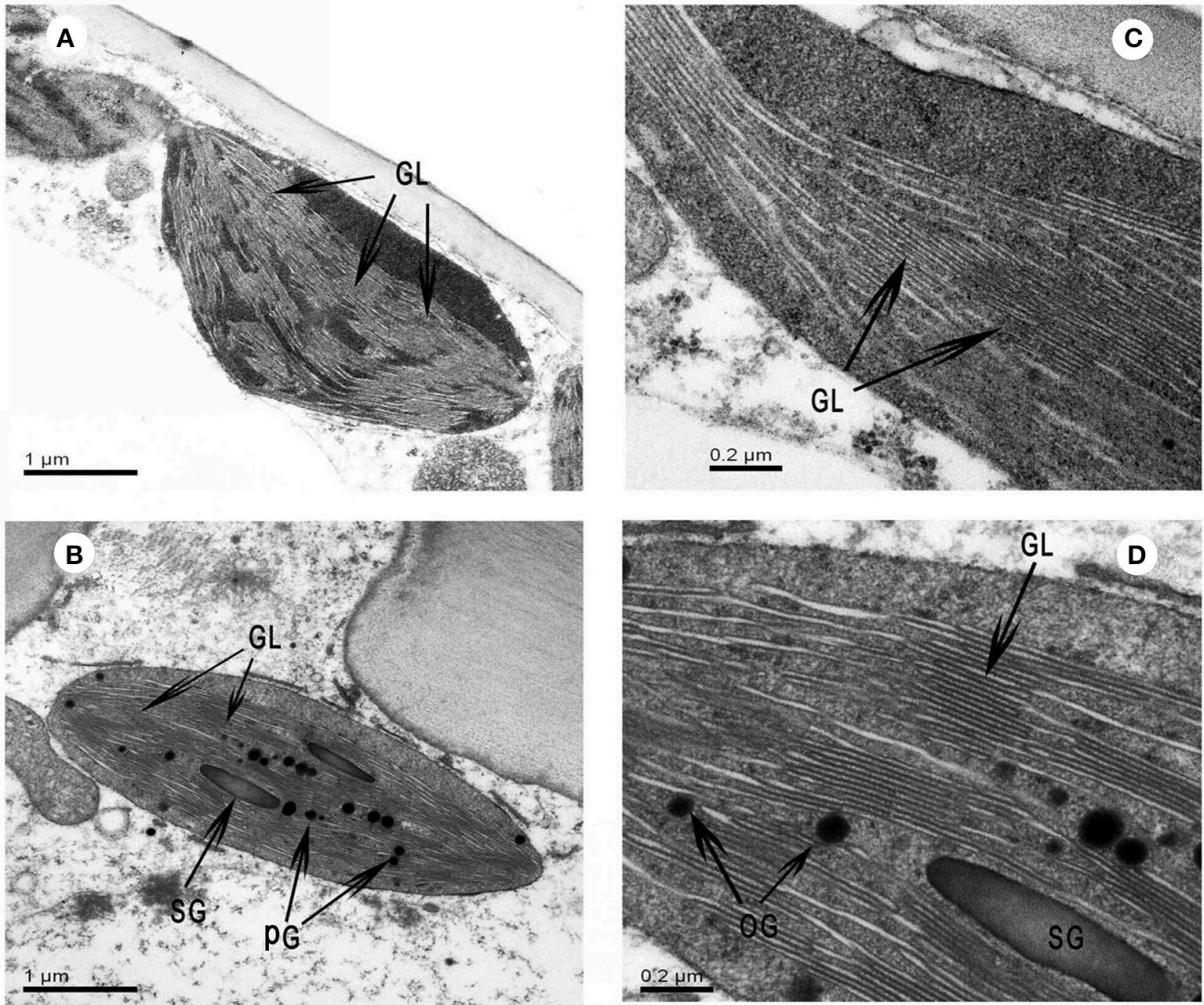
The chloroplast ultrastructure in leaf **(A,B)** and sarcotesta **(C,D)** of *Torreya grandis* cv. “Merrillii.” The samples for microscopy were taken on 5th July in 2015, 81 days after seed protrusion (DASP = 81 days). PG, plastoglobuli; GL, grana lamellae; SG, starch grains.

The chloroplast ultrastructure was examined on one occasion at 81 DASP in 2015. Chloroplasts were spherical both in the leaves and in the sarcotesta (Figure 9). There was no significant difference in the chloroplast length, width, or grana number between leaves and sarcotesta (Table 2); however, the granal lamellae number was significantly greater in sarcotesta than in leaves (*P* < 0.05; Figure 9 and Table 2). Leaves at 81 DASP possessed well-differentiated chloroplasts that contained numerous layers of grana and well-developed stromal lamellae (Figures 9A,B). The system of granal and intergranal thylakoids was fully developed also in seeds, accompanied by many plastoglobuli and starch grains in the chloroplasts of sarcotesta (Table 2, Figures 9C,D). However, no plastoglobuli or starch grains were observed in the chloroplasts in leaves (Figures 9A,B).

**Table 2:** Chloroplast characteristics in leaves and sarcotesta of *Torreya grandis* cv. “Merrillii” at 81 days after seed protrusion in 2015 (DASP = 81 days).

**Parameters**	**Leaf**	**Sarcotesta**
Grana number	19.5 ± 2.5^a^	21.8 ± 1.10^a^
Grana lamellae number	7.8 ± 0.33^b^	9.28 ± 0.48^a^
Osmiophilic globule number	0^b^	21.0 ± 2.16^a^
Osmiophilic globule size (μm^2^)	na^*^	0.009 ± 0.002^a^
Starch grains number	0^b^	1.25 ± 0.43^a^
Starch grains size (μm^2^)	na^*^	0.12 ± 0.03^a^

* *na (not applicable), because there were no osmiophilic globules or starch grains in the leaf chloroplasts. Data are mean ± SD. Different letters denote significant differences at P ≤ 0.05 level. n = 3 trees*.

### Effects of DCMU incubation on the seed dry weight and seed oil quality

The photosynthetic contribution of seeds to the seed weight was assessed by measuring the reduction in their weight after DCMU application (Table 3). This was carried out separately with intact seeds, and with seeds where the sarcotesta were removed before measuring the weight. The fresh weight of the intact seeds and seeds with sarcotesta removed was reduced by 22.0 and 5.4% from that of the control seeds, respectively. The contribution of seed photosynthesis to the dry weight of the seeds, as estimated by the reduction in the dry weight caused by the DCMU application, was in the intact seeds and seeds with sarcotesta removed about 25.4 and 40.0%, respectively (Table 3). The DCMU application significantly reduced also the length and width of the seeds. In addition to reducing the size and weight of the seeds, the DCMU application significantly reduced the oil content of the seeds by about 25.5% as compared with the control seeds (Table 3).

**Table 3:** Changes in *Torreya grandis* cv. “Merrillii” seed characters caused by treatment with 3-(3,4-dichlorophenyl)-1,1-dimethylurea (DCMU) in 2015.

**Parameter**	**Control**	**DCMU**
Length (mm)	30.00 ± 0.30^a^	28.52 ± 1.40^b^
Width (mm)	21.34 ± 0.83^a^	19.89 ± 0.48^b^
Fresh weight of intact seeds (g)	8.55 ± 0.50^a^	6.67 ± 0.21^b^
Fresh weigh of seeds with sarcotesta removed (g)	2.60 ± 0.13^a^	2.46 ± 0.19^a^
Dry weight of intact seeds (g)	2.28 ± 0.14^a^	1.70 ± 0.19^b^
Dry weight of seeds with sarcotesta removed (g)	1.80 ± 0.07^a^	1.08 ± 0.16^b^
Oil content (%)	54.1 ± 0.5^a^	40.3 ± 0.06^b^

*Data are mean ± SD. Different letters denote significant differences at P ≤ 0.05 level. n = 5 trees*.

DCMU application changed the oil composition of the seeds. The statistically significant percentage changes in the contents of the fatty acids were as follows (Table 4): Octadecanoic acid (C18:0) and oleic acid (C18:1) contents decreased significantly by 12% (from 2.5 to 2.2%) and by 15% (from 31.8 to 27.1%), respectively. Cetylic acid (C16:0), linoleic acid (C18:2Δ^9C, 12C^), linolenic acid (C18:3Δ^9C, 12C, 15C^) and sciadonic acid (C20:3Δ^5C, 11C, 14C^) contents increased significantly by 6, 4, 16.7, and 25.5%, respectively (Table 4). DCMU application also slightly affected the total contents of saturated fatty acids and unsaturated fatty acids (Table 4).

**Table 4:** Changes in *Torreya grandis* cv. “Merrillii” seed oil composition caused by treatment with 3-(3,4-dichlorophenyl)-1,1-dimethylurea (DCMU) in 2015.

**Parameter**	**Control**	**DCMU**
Palmitic acid (C16:0)	6.8 ± 0.06^b^	7.2 ± 0.02^a^
Stearic acid (C18:0)	2.5 ± 0.01^a^	2.3 ± 0.02^b^
Oleic acid (C18:1Δ^11C^)	31.8 ± 0.04^a^	27.1 ± 0.07^b^
Linoleic acid (C18:2Δ^9C, 12C^)	44.1 ± 0.07^b^	45.9 ± 0.12^a^
Linolenic acid (C18:3Δ^9C, 12C, 15C^)	0.4 ± 0.00^b^	0.5 ± 0.00^a^
Arachidic acid (C20:0)	0.7 ± 0.01^a^	0.7 ± 0.01^a^
Eicosaenoic acid (C20:1Δ^11C^)	0.6 ± 0.01^a^	0.6 ± 0.02^a^
Eicosadienoic acid (C20:2Δ^11C, 14C^)	2.5 ± 0.00^a^	2.6 ± 0.04^a^
Sciadonic acid (C20:3Δ^5C, 11C, 14C^)	10.6 ± 0.07^b^	13.3 ± 0.05^a^
Saturated fatty acid	10.0 ± 0.11^a^	10.1 ± 0.06^a^
Unsaturated fatty acid	90.0 ± 0.11^a^	89.1 ± 0.06^a^

*The data indicate the contents of the various fatty acids as percentages in oil content*.

*Data are mean ± SD. Different letters denote significant differences at P ≤ 0.05 level. n = 3 trees*.

## Discussion

### Seed development in *Torreya*

The dry/fresh weight ratio of fruits can be used to assess the different phases of fruit growth (Imai and Ogawa, 2009). In the present study, seed development was divided into two phases according to the seed growth pattern. The first phase was the rapid seed growth stage, which was characterized by a rapid increase in seed size. This occurred from 1 to 69 DASP in 2015 and from 1 to 46 in 2016 (Figure 2). During this period, the seed dry weight/fresh weight ratio decreased to its lowest value (Figures 3C,F), indicating that seed water content increased. In the second phase (the seed filling stage), the increase of seed length and width slowed as seed dry weight continued to increase. The main oil synthesis was obviously during this period, as suggested by the result of Tian et al. (1989). They found that the oil content in *Torreya* seeds rapidly increased from 21.4 to 54.5% during the period from 5th July to 5th September.

### Photosynthetic characteristics in *Torreya* seed sarcotesta

The seeds had in the present study relatively high light saturation point LSP the period from 27 to 91 DASP; however, the LSP decreased rapidly from 92 to 106 DASP (Figure 4A). The site where the *Torreya* cultivar was studied is in Southeastern China, having both high light intensity and high air temperature during the summer months from July to August, corresponding to the period from 75 to 138 DASP in this study. Thus, during the second stage of seed development, the seeds may have been exposed to an excess light which cannot be used for photosynthesis, so that and their PSII were then susceptible to photoinhibition owing to the decreased LSP (Galmés et al., 2007). The photoinhibition of PSII can be detected *in vivo* by a significant decrease in the *F*_*v*_*/F*_*m*_ (Krause and Weis, 1991). However, no change in *F*_*v*_*/F*_*m*_ was observed in seed sarcotesta of the *Torreya* cultivar between 91 and 106 DASP (Table 1), indicating that the photoinhibition did not occur in the sarcotesta during this period. However, the Φ_*PSII*_ was significantly lower at 106 DASP than that at 91 DASP. This is consistent with the findings of Zhang et al. (2011) who reported that soybean leaves dissipate the excess excitation energy thermally by the down-regulation of PSII activity to protect their photosynthetic apparatus from photodamage under drought condition. The parameter *qP* indicates the proportion of PSII reaction centers that are open (Maxwell and Johnson, 2000). Since Φ_*PSII*_ = *qP*×*F*_*v*_'*/F*_*m*_', the significant reduction of Φ_*PSII*_ found in the present study from 91 to 106 DASP resulted primarily from an increased proportion of closed PSII reaction centers, as indicated by the lower values of *qP*; while values of *F*_*v*_'*/F*_*m*_' were essentially the same during that period (Table 1). These results suggest that large proportion of the PSII reaction centers were closed at 106 DASP so that they may have operated as quenchers to help PSII from photoinhibition (Huner et al., 2006).

There is another possible reason for the significant reduction of LSP during 91 to 106 DASP, while with little change in *F*_*v*_*/F*_*m*_ of the seed sarcotesta. Plant may decrease photosynthetic rate to re-balance the source-sink ratio when carbohydrate levels reached a threshold value in leaves (Paul and Foyer, 2001). Accordingly, we suggest that the size growth of the seeds (storage organ) was close to completion during that period from 91 to 106 DASP (Figure 2): so that more carbohydrate accumulation in seed sarcotesta may have taken place. That may have caused the reduction of photosynthetic rate and LSP. To test this hypothesis, further studies are necessary to investigate the trend of carbohydrate content in sarcotesta during the seed development.

Furthermore, the apparent quantum use efficiency (AQE) was between 0.04 and 0.06 mol CO_2_ (mol photons)^−1^ in the first stage of seed development (from 1 to 69 DAPS, Figure 4B). These values of AQE are higher than those observed earlier the *Torreya* leaves associated to the seeds (0.01–0.02 mol CO_2_ mol^−1^, Huang, 2015). Similar results of higher AQE in capsule wall compared with its associated leaves has also been observed in cotton (Hu, 2013). These results suggest that the seed sarcotesta can effectively use low light for photosynthesis, especially in the early morning or late afternoon, which is important for yield formation.

Respiration provide the energy for the synthesis of new phytomass (Amthor, 2000). In the present study, the rate of dark respiration *R*_*d*_ calculated per seed dry weight was very high at the initial stage of seed development, then *R*_*d*_ rapidly decreased. These changes in the rate of dark respiration are consistent with the rate of increase in the seed dry mass (Figures 3B,E), supporting the notion of respiratory cost of synthesis of new biomass.

The *P*_(*g,max*)_ expressed per unit surface area significantly decreased by 18.7% between 27 and 91 DASP (Figure 4C); however, the *P*_(*g,max*)_ per seed dramatically increased during the same time (Figure 4D). This may be due to a rapid increase in the surface area of the seeds (Figure 2C). The seed area significantly increased by 18.2% also later from 91 to 138 DASP; however, the *P*_(*g,max*)_ per surface area significantly decreased during the same time by 49%. Thus, a significant decrease in the *P*_(*g,max*)_ per seed during the 91 to 138 DASP stage can be attributed to the decreased photosynthetic rate per seed surface area.

Chlorophyll plays an important role in the light absorption and energy transduction, which are the basis of photosynthesis. In the present study, the initial rapid decrease in chlorophyll content per area from 27 to 69 DASP (Figure 5) could have been caused to a large extent by a dilution effect due to the fast increase in seed volume. There was some increase in the chlorophyll content from 91 to 106 DASP, when the *P*_(*g,max*)_ per surface area decreased by 22.7%. These findings indicate that the decrease of seed photosynthesis during the period from 91 to 106 DASP was not caused by low chlorophyll content. This notion is in agreement with the general observation that light-saturated photosynthesis of leaves is largely unrelated to their chlorophyll content (Björkman, 1981).

However, the Chl_a+b_ content significantly decreased by 23% accompanied by significantly decreased *F*_*v*_*/F*_*m*_ form 106 to 138 DASP (Table 1 and Figure 5). The Chl_a/b_ ratio was significantly higher at 138 DASP than that at 106 DASP (Figure 5), indicating that degradation of photosynthetic pigments, especially Chl_b_ took place during that period. Therefore, the photosynthetic apparatus seemed to begin to be damaged at the late second development stage of the seeds.

Nitrogen may be a key regulator of photosynthesis in plants. In leaves, more than half of the nitrogen is allocated to photosynthetic proteins, such as Rubisco (Evans, 1989), and the amount, or activity, of Rubsico correlates strongly with photosynthetic rate (Evans, 1989; Reich and Walters, 1994; Priwitzer et al., 1998; Hrstka et al., 2005). Thus, nitrogen content has been used as a qualitative measure of the Rubisco content (Makino, 2003). In the present study, the Rubisco activity and nitrogen content of sarcotesta both showed decreasing trend during seed development (Figures 6A, 7A). Moreover, a positive correlation was found between Rubisco activity and *P*_(*g,max*)_ per surface area (Figure 6A). The nitrogen content in sarcotesta significantly decreased by 25% from 106 to 138 DASP (Figure 7A). We also observed a significant positive correlation between the *P*_(*g,max*)_ calculated per dry weight and nitrogen content in the sarcotesta (Figure 7B). Therefore, the decreased Rubisco activity caused by the decreased nitrogen content may have caused the damage of the photosynthetic apparatus. Liu et al. (2014) reported that the nitrogen content in *Torreya* kernels significantly increased by 50% from 19 July to 19 August, corresponding to 95 ~ 126 DASP in present study. It has been reported that nitrogen in foliage and pericarp of almond is rapidly imported into the embryo (Weinbaum and Muraoka, 1986). Similarly, the decrease in percent nitrogen in sarcotesta in the present study suggests that the amount of nigtrogen-containing compounds, such as proteins or amino acids, decreased, as they were rapidly imported into the developing seed.

### The relative contribution of seed photosynthesis to seed development

The photosynthetic rate per seed area significantly decreased from 32 to 69 DASP, which corresponds to the first stage of seed development between 17th May and 23rd June, but was then significantly higher than the rate in the associated current- year leaves which began to sprout 30th April and reached the maximum rate of photosynthesis 20th July. Thus, we suggest that the photosynthesis in seeds plays an important role in seed growth during the first stage of seed development. However, at the second stage of seed development, when seed size growth is close to completion, the constant photosynthetic rate in the associated leaves, accompanied by the significantly decreased photosynthetic rate in seed sarcotesta during that time (Figure 8), suggest a less important role of seed photosynthesis on dry matter accumulation during that period. The chloroplasts of leaves possessed many well-organized grana thylakoids with no plastoglobuli or starch grains, while those in sarcotesta contained a large number of granas accompanied by many plastoglobuli and starch grains (Figure 9). Plastoglobuli are usually formed in senescing chloroplasts (Lichtenthaler, 2013). Our observation of these characteristics indicates that the chloroplasts of sarcotesta began to senesce in the second stage of seed development. In addition, the accumulated starch grains can dislocate the grana (Bondada and Oosterhuis, 2003) and push them toward the periphery of the chloroplast, which might impede photosynthetic activity.

The import of carbon from photosynthetically active source tissues is needed for seed development (Gallardo et al., 2008), and it also affects the synthesis of fatty acids (Xiong et al., 2010). Rolletscheck et al. (2005) have shown that illumination of developing soybean seeds causes photosynthetic release of significant amounts of O_2_. This seed photosynthesis plays a key role in the local energy state, storage metabolism and flux of carbon to lipid biosynthesis. Although green leaves are the main sources of photosynthate production, sarcotesta photosynthesis was also supplies assimilates for oil synthesis. Indeed, our results shows that the reduction in dry weight and oil content of the intact seed induced by DCMU application was 25.4 and 25.5%, respectively (Table 3). This indicates that the reduced supply of seed sarcotesta photosynthates caused by the DCMU application inhibited oil synthesis. Results from earlier studies suggests that the contents of polyunsaturated fatty acids are significantly increased as a response to abiotic stresses, such as freezing stress, salt stress, oxidative stress etc. (Watanabe et al., 2004; Zhou et al., 2010; Zhang et al., 2012). Considering that DCMU application inhibits the photosynthetic electron transport, the increased polyunsaturated fatty acids content caused by DCMU application can be explained as an adaptive response to the deficiency of the photosynthate supply from seed photosynthesis. This is in line with Wang et al. (2016) who found that green siliques photosynthesis greatly influences seed oil content and oil composition of oilseed rape.

## Conclusions

Our results show that seed photosynthesis is important for the biomass accumulation and oil synthesis in *Torreya grandis* cv. “Merrillii” seeds. The seed photosynthesis is especially active during the first stage of seed development, when the photosynthetic production in the attached current-year leaves is much lower. It appears that the down-regulation of PSII activity, of Rubisco activity, and of Chl_(a+b)_, and nitrogen contents in the sarcotesta are well coordinated in order to re-balance the source-sink ratio at the late second stage of seed development. Simultaneously the photosynthetic activity of the attached current year leaves increases, so that the photosynthetic activity is higher in the leaves than in the seeds during the second stage of seed development. Our results also show that seed photosynthesis inhibition affects the fatty acid composition of the seeds. These results have implications for cultivation practices in the future, as high oil content require efficient photosynthesis during the seed development stage of the *Torreya* cultivar. Therefore, the high photosynthetic activity in sarcotesta may be a useful trait for the selection of cultivars with high yield and high oil contents.

## Author contributions

Designing the work: YH, JW; running the experiments: YH, YZ, WY, XD; data analysis and statistics: YH, YZ; article writing and revising: YH, YZ, HH, LS, RZ, JW.

### Conflict of interest statement

The authors declare that the research was conducted in the absence of any commercial or financial relationships that could be construed as a potential conflict of interest. The reviewer DA and handling Editor declared their shared affiliation.

## Supplementary material

Figure S1 The standard gas chromatogram of the main fatty acids in seeds of *Torreya grandis* cv. “Merrillii.”

Figure S2 Light responses of net photosynthesis *P*_n_ in seeds of *Torreya grandis* cv. “Merrillii” during seed development; expressed on surface area **(A)**, dry weight **(B)**, and single seed **(C)** basis. For the P_n_ values mean ± SD is indicated. Different letters denote significant differences at *P* ≤ 0.05 level. *n* = 4. The light responses were measured on eight dates indicated by the symbols and DASP values in the upper right-hand corner of the uppermost panel. DASP, days after seeds protrusion. Note: The values of *P*_*n*_ are negative because of high rates of respiration. The rates were high because the measurements were carried out with intact seed containing also the non-photosynthetic embryo in addition to the photosynthetic sarcotesta. The absolute value of the negative *P*_*n*_ indicates the re-fixation by the sarcotesta photosynthesis of the CO_2_ released from respiration. See introduction of the main text for further explanation.

Table S1 The inhibitive effect of different concentrations of DCMU on the ETR of seed in comparison with the control.

## References

[B1] Allen D. K. Ohlrogge J. B. Shachar-Hill Y. (2009). The role of light in soybean seed filling metabolism. Plant J. 58, 220–234. 10.1111/j.1365-313X.2008.03771.x 19077167

[B2] Allen J. F. Holmes N. G. (1986). Electron transport and redox titration in Photosynthesis Energy Transduction, a Practical Approach, eds Hipkins M. F. Baker N. R. (Oxford: IRL Press), 103–141.

[B3] Amthor J. S. (2000). The McCree-de Wit-Penning de Vries-Thornley respiration paradigms: 30 years later. Ann. Bot. 86, 1–20. 10.1006/anbo.2000.1175

[B4] Aschan G. Pfanz H. (2003). Non-foliar photosynthesis: a strategy of additional carbon acquisition. Flora 198, 81–97. 10.1078/0367-2530-00080

[B5] Asokanthan P. S. Johnson R. W. Griffith M. Krol M. (1997). The photosynthetic potential of canola embryos. Physiol. Plantarum 101, 353–360. 10.1111/j.1399-3054.1997.tb01008.x

[B6] Baryeh E. A. (2001). Physical properties of bambara groundnuts. J. Food Eng. 47, 321–326. 10.1016/S0260-8774(00)00136-9

[B7] Baud S. Lepiniec L. (2010). Physiological and developmental regulation of seed oil production. Prog. Lipid Res. 49, 235–249. 10.1016/j.plipres.2010.01.001 20102727

[B8] Björkman O. (1981). Responses to different quantum flux densities, in Physiological Plant Ecology I, eds Lange O. L. Nobel P. S. Osmond C. B. Ziegler H. (Berlin; Heidelberg: Springer), 57–107.

[B9] Blanke M. M. Lenz F. (1989). Fruit photosynthesis. Plant Cell Environ. 12, 31–36. 10.1111/j.1365-3040.1989.tb01914.x

[B10] Bondada B. R. Oosterhuis D. M. (2003). Morphometric analysis of chloroplast of cotton leaf and fruiting organs. Biol. Plantarum 47, 281–284. 10.1023/B:BIOP.0000022266.67097.3d

[B11] Charles - Edwards D. A. (1981). The mathematics of photosynthesis and productivity. Q. Rev. Biol. 4, 304.

[B12] Chen L. S. Cheng L. (2003). Carbon assimilation and carbohydrate metabolism of ‘Concord’ grape (*Vitis labrusca* L.) leaves in response to nitrogen supply. J. Am. Soc. Hortic. Sci. 128, 754–760.

[B13] Chen L. S. Cheng L. L. (2007). The sun-exposed peel of apple fruit has a higher photosynthetic capacity than the shaded peel. Funct. Plant Biol. 34, 1038–1048. 10.1071/FP07111 32689432

[B14] Chow W. S. Hope A. B. Anderson J. M. (1990). A reassessment of the use of herbicide binding to measure photosystem II reaction centres in plant thylakoids. Photosynth. Res. 24, 109–113. 10.1007/BF00032650 24419771

[B15] Dai W. S. Li Z. J. Huang J. Q. Zhang M. Yu W. W. Chen X. J. (2008). A Standard of Forestry Sector of P.R. China: Technical Regulation for Torreya grandis cv. “Merrillii” Production. LY/T 1774-2008. Available online at: http://www.doc88.com/p-7919531357665.html

[B16] Dejong T. M. Walton E. F. (1989). Carbohydrate requirements of peach fruit growth and respiration. Tree Physiol. 5, 329–335. 10.1093/treephys/5.3.329 14972978

[B17] Evans J. R. (1989). Photosynthesis and nitrogen relationships in leaves of C_3_ plants. Oecologia 78, 9–19. 10.1007/BF00377192 28311896

[B18] Fan D. Y. Nie Q. Hope A. B. Hillier W. Pogson B. J. Chow W. S. (2007). Quantification of cyclic electron flow around Photosystem I in spinach leaves during photosynthetic induction. Photosynth. Res. 94, 347–357. 10.1007/s11120-006-9127-z 17211579

[B19] Furbank R. T. White R. Palta J. A. Turner N. C. (2004). Internal recycling of respiratory CO_2_ in pods of chickpea (*Cicer arietinum* L.): the role of pod wall, seed coat, and embryo. J. Exp. Bot. 55, 1687–1696. 10.1093/jxb/erh190 15234993

[B20] Gallardo K. Thompson R. Burstin J. (2008). Reserve accumulation in legume seeds. C. R. Biol. 331, 755–762. 10.1016/j.crvi.2008.07.017 18926489

[B21] Galmés J. Abadía A. Medrano H. Flexas J. (2007). Photosynthesis and photoprotection responses to water stress in the wild - extinct plant *Lysimachia minoricensis*. Environ. Exp. Bot. 60, 308–317. 10.1016/j.envexpbot.2006.12.016

[B22] Genty B. Briantais J. M. Baker N. R. (1989). The relationship between the quantum yield of photosynthetic electron transport and quenching of chlorophyll fluorescence. BBA Gen Subjects 990, 87–92. 10.1016/S0304-4165(89)80016-9

[B23] Goffman F. D. Alonso A. P. Schwender J. Shachar-Hill Y. Ohlrogge J. B. (2005). Light enables a very high efficiency of cabon storage in developing embryos of rapeseed. Plant Physiol. 138, 2269–2279. 10.1104/pp.105.063628 16024686 PMC1183413

[B24] Hieke S. Menzel C. M. Lüdders P. (2002). Shoot development, chlorophyll, gas exchange and carbohydrates in lychee seedlings (*Litchi* chinensis). Tree Physiol. 22, 947–953. 10.1093/treephys/22.13.947 12204851

[B25] Hiratsuka S. Suzuki M. Nishimura H. Nada K. (2015). Fruit photosynthesis in Satsuma mandarin. Plant Sci. 241, 65–69. 10.1016/j.plantsci.2015.09.026 26706059

[B26] Hrstka M. Urban O. Marek M. V. (2005). Long-term effect of elevated CO_2_ on spatial differentiation of ribulose-1,5-bisphosphate carboxylase/oxygenase activity in Norway spruce canopy. Photosynthetica 43, 211–216. 10.1007/s11099-005-0035-9

[B27] Hu Y. Y. (2013). Photosynthetic Characteristics and Strategies of Acclimation of Non-foliar Organs in Cotton (Gossypium spp.) Respond to Water Deficit [D]. Shihezi: Shihezi University (in Chinese).

[B28] Hu Y. Y. Oguchi R. Yamori W. von Caemmerer S. Chow W. S. Zhang W. F. (2013). Cotton bracts are adapted to a microenvironment of concentrated CO_2_ produced by rapid fruit respiration. Ann. Bot. 112, 31–40. 10.1093/aob/mct091 23625144 PMC3690982

[B29] Hu Y. Y. Zhang Y. L. Luo H. H. Li W. Oguchi R. Fan D. Y.. (2012). Important photosynthetic contribution from the non-foliar green organs in cotton at the late growth stage. Planta 235, 325–336 10.1007/s00425-011-1511-z 21904871

[B30] Hua W. Li R. J. Zhan G. M. Liu J. Li J. Wang X. F.. (2012). Maternal control of seed oil content in *Brassica napus*: the role of silique wall photosynthesis. Plant J. 69, 432–444. 10.1111/j.1365-313X.2011.04802.x 21954986

[B31] Huang Z. G. (2015). The Study of Photosynthetic Physiological Characteristics in The Developmental Process in Leaves of Torreya grandis [D]. Hangzhou: Zhejiang A&F University (in Chinese).

[B32] Huner N. P. A. Ivanov A. G. Sane P. V. Pocock T. Krói M. Balseris A. (2006). Photoprotection of photosystem II: reaction center quenching versus antenna quenching in Photoprotection, Photoinhibition, Gene Regulation, and Environment, eds Demmig B. Adams W. W. III Mattoo A. K. (Dordrecht: Springer Netherlands), 155–173.

[B33] Imai S. Ogawa K. (2009). Quantitative analysis of carbon balance in the reproductive organs and leaves of *Cinnamomum camphora* (L.). Presl. J. Plant Res. 122, 429–437. 10.1007/s10265-009-0233-9 19396511

[B34] King S. P. Badger M. R. Furbank R. T. (1998). CO_2_ refixation characteristics of developing canola seeds and silique wall. Aust. J. Plant Physiol. 25, 377–386. 10.1071/PP97157

[B35] Kitajima M. Butler W. L. (1975). Quenching of chlorophyll fluorescence and primary photochemistry in chloroplasts by dibromothymoquinone. Biochim. Biophys. Acta 376, 105–115. 10.1016/0005-2728(75)90209-1 1125215

[B36] Krause G. H. Weis E. (1991). Chlorophyll fluorescence and photosynthesis: the basics. Annu. Rev. Plant Biol. 42, 301–313. 10.1146/annurev.pp.42.060191.001525

[B37] Larcher W. (2003). Physiological Plant Ecology and Stress Physiology of Functional Groups, 4th Edn. Berlin: Springer-Verlag.

[B38] Li X. J. Hou J. H. Bai K. Z. Yang X. H. Lin J. X. Li Z. S. (2004). Activity and distribution of carbonic anhydrase in leaf and ear parts of wheat (*Triticum aestivum* L.). Plant Sci. 166, 627–632. 10.1016/j.plantsci.2003.10.031

[B39] Li X. J. Wang H. G. Li H. B. Zhang L. Y. Teng N. J. Lin Q. Q. (2006). Awns play a dominant role in carbohydrate production during the grain-filling stages in Wheat (*Triticum aestivum)*. Physiol. Plantarum 127, 701–709. 10.1111/j.1399-3054.2006.00679.x

[B40] Lichtenthaler H. K. (1987). Chlorophylls and carotenoids: pigments of photosynthetic biomembranes. Method. Enzymol. 148, 350–382. 10.1016/0076-6879(87)48036-1

[B41] Lichtenthaler H. K. (2013). Plastoglobuli, thylakoids, chloroplast structure and development of plastids in Plastid Development in Leaves During Growth and Senescence Advances in Photosynthesis and Respiration, eds Biswal B. Krupinska K. Biswal U. C. (Berlin: Springer Press), 337–361.

[B42] Liu M. M. Zeng Y. R. Jiang J. B. Zhang K. L. Yu W. W. (2014). Mineral elements in leaves and seeds of *Torreya grandis* ‘Merrillii’ during seed development. Nonwood Forest Res. 2, 105–109. 10.14067/j.cnki.1003-8981.2014.02.017

[B43] Liu Z. M. Zhao H. B. Huang Y. J. Mei L. Huang J. Q. Hu Y. (2017). Histological studies on megagametophyto and embrygeny development. J. Fruit Sci. 2, 231–237. 10.13925/j.cnki.gsxb.20160206

[B44] Lytovchenko A. Eickmeier I. Pons C. Osorio S. Szecowka M. Lihmberg K.. (2011). Tomato fruit photosynthesis is seemingly unimportant in primary metabolism and ripening but plays a considerable role in seed development. Plant Physiol. 157, 1650–1663. 10.1104/pp.111.186874 21972266 PMC3327185

[B45] Makino A. (2003). Rubisco and nitrogen relationships in rice:leaf photosynthesis and plant growth. Soil Sci. Plant Nutr. 49, 319–327. 10.1080/00380768.2003.10410016

[B46] Martinez D. E. Luquez V. M. Bartoli C. G. Guiamé J. J. (2003). Persistence of photosynthetic components and photochemical efficiency in ears of water-stressed wheat (*Tritium aestinvum)*. Physiol. Plantarum 119, 519–525. 10.1046/j.1399-3054.2003.00195.x

[B47] Maxwell K. Johnson G. N. (2000). Chlorophyll fluorescence - a practical guide. J. Exp. Bot. 51, 659–668. 10.1093/jexbot/51.345.659 10938857

[B48] Maxwell R. J. Marmer W. N. (1983). Fatty acid analysis: phospholipid- rich analysis. Lipids 18, 453–459. 10.1007/BF02535785 6621256

[B49] Maydup M. L. Antonietta M. Guiamet J. J. Graciano C. López J. R. Tambussi E. A. (2010). The contribution of ear photosynthesis to grain filling in bread wheat (*Triticum aestivum* L.). Field Crop Res. 119, 48–58. 10.1016/j.fcr.2010.06.014

[B50] O'Neil C. E. Keast D. R. Nicklas T. A. (2012). Out-of-hand nut consumption is associated with improved nutrient intake and health risk markers in US children and adults: National Health and Nutrition Examination Survey 1999-2004. Nutr. Res. 32, 185–194. 10.1016/j.nutres.2012.01.005 22464805

[B51] Paul M. J. Foyer C. H. (2001). Sink regulation of photosynthesis. J. Exp. Bot. 52, 1383–1400. 10.1093/jexbot/52.360.1383 11457898

[B52] Platt-Aloia K. A. Thompson W. W. (1981). Ultrastructure of the mesocarp of mature avocado fruit and change associated with ripening. Ann. Bot. 48, 451–465. 10.1093/oxfordjournals.aob.a086149

[B53] Prebeg T. Wrischer M. Fulgosi H. Ljubešić N. (2008). Ultrastructural characterization of the reversible differentiation of chloroplasts in cucumber fruit. J. Plant Biol. 51, 122–131. 10.1007/BF03030721

[B54] Priwitzer T. Urban O. Šprtová M. Marek M. V. (1998). Chloroplastic carbon dioxide concentration in Norway spruce [*Picea Abies* (L.). Karst.] needles relates to the position within the crown. Photosynthetica 35, 561–571. 10.1023/A:1006983008272

[B55] Proietti P. Famiani F. Tombesi A. (1999). Gas exchange in olive fruit. Photosynthetica 36, 423–432. 10.1023/A:1007028220042

[B56] Quebedeaux B. Chollet R. (1975). Growth and development of soybean (*Glycine-max* [L] merr). pods - CO_2_ exchange and enzyme studies. Plant Physiol. 55, 745–748. 10.1104/pp.55.4.745 16659160 PMC541699

[B57] Ranjan S. Singh R. Soni D. K. Pathre U. V. Shirke P. A. (2012). Photosynthetic performance of *Jatropha curcas* fruits. Plant Physiol. Biochem. 52, 66–76. 10.1016/j.plaphy.2011.11.008 22305068

[B58] Reich P. B. Walters M. B. (1994). Photosynthesis-nitrogen relations in Amazonian tree species. II. variation in nitrogen vis-a-vis specific leaf-area influences mass - based and area-based expressions. Oecologia 97, 73–81. 10.1007/BF00317910 28313591

[B59] Rolletscheck H. Radchuk R. Klukas C. Schreiber F. Wobus U. Borisjuk L. (2005). Evidence of a key role for photosynthetic oxygen release in oil storage in developing soybean seeds. New Phytol. 167, 777–786. 10.1111/j.1469-8137.2005.01473.x 16101914

[B60] Ruuska S. A. Schwender J. Ohlrogge J. B. (2004). The capacity of green oilseeds to utilize photosynthesis to drive biosynthetic processes. Plant Physiol. 136, 2700–2709. 10.1104/pp.104.047977 15347783 PMC523334

[B61] Schreiber U. Bilger W. Neubauer C. (1994). Chlorophyll fluorescence as a nonintrusive indicator for rapid assessment of *in vivo* photosynthesis, in Ecophysiology of Photosynthesis, eds Schulze E. D. Caldwell M. M. (Berlin: Springer Press), 49–70.

[B62] Schuman G. E. Stanley M. A. Knudsen D. (1972). Automated total nitrogen analysis of soil and plant samples. Soil Scie. Soc. Am. J. 37, 480–481. 10.2136/sssaj1973.03615995003700030045x

[B63] Schwender J. Goffman F. Ohlrogge J. B. Shachar-Hill Y. (2004). Rubisco without the Calvin cycle improves the carbon efficiency of developing green seeds. Nature 432, 779–782. 10.1038/nature03145 15592419

[B64] Singal H. R. Gurmeet T. Anita D. Randhir S. (1995). Pod photosynthesis and seed dark CO_2_ fixation support oil synthesis in developing Brassica seeds. J. Biosciences 20, 49–58. 10.1007/BF02711580

[B65] Tian J. X. Wu M. C. Zhong S. M. (1989). Physical and chemical analysis of the seed of *Torreya grands* Fort. J. ZJ. For. Coll. 6, 13–19.

[B66] Wang C. L. Hai J. B. Yang J. L. Tian J. H. Chen W. J. Chen T. (2016). Influence of leaf and siloque phtosynthesis on seeds yield and seeds oil quality of oilseed rape (*Brassica napus* L.). Eur. J. Agron. 74, 112–118. 10.1016/j.eja.2015.12.008

[B67] Wang X. Y. Xiu L. L. (2005). The nutritional and functional component in *Torreya grandis* review. Food Res. Exploit. 26, 20–22. 10.3969/j.issn.1005-6521.2005.02.007

[B68] Watanabe K. Oura T. Sakai H. (2004). Yeast Δ12 fatty acid desaturase: gene cloning, expression, and function. Biosci. Biotechnol. Biochem. 68, 721–727. 10.1271/bbb.68.721 15056908

[B69] Weinbaum S. A. Muraoka T. T. (1986). Nitrogen redistribution from almond foliage and pericarp to the almond embryo. J. Am. Soc. Hortic. Sci. 111, 224–228.

[B70] Whiley A. W. Schaffer B. Lara S. P. (1992). Carbon dioxide exchange of developing avocado (*Persea americana* Mill.) fruit. Tree Physiol. 11, 85–94. 10.1093/treephys/11.1.85 14969969

[B71] Wullschleger S. D. Oosterhuis D. M. (1990). Photosynthetic and respiratory activities of fruiting forms within the cotton canopy. Plant Physiol. 94, 463–469. 10.1104/pp.94.2.463 16667734 PMC1077254

[B72] Xiong W. Gao C. Yan D. Wu C. Wu Q. (2010). Double CO_2_ fixation in photosynthesis-fermentation model enhances algal lipid synthesis for biodiesel production. Bioresour. Technol. 101, 2287–2293. 10.1016/j.biortech.2009.11.041 19963369

[B73] Xu H. L. Gauthier L. Desjardins Y. Gosselin A. (1997). Photosynthesis in leaves, fruits, stem and petioles of greenhouse-grown tomato plants. Photosynthetica 33, 113–123. 10.1023/A:1022135507700

[B74] Xu Q. Y. Wu J. F. Cao Y. R. Yang X. Y. Wang Z. J. Huang J. Q. (2016). Photosynthetic characteristics of leaves and fruits of Hickory (*Carya cathayensis*, Sarg.) and Pecan (*Carya illinoensis*, K.Koch) during fruit development stages. Trees 30, 1523–1534. 10.1007/s00468-016-1386-5

[B75] Yu X. Y. Li P. Dong X. Y. Dong L. M. Shu S. M. (1986). Aril structure and its aromatic oil in *Torreya grandis* Fort.ex Lindl. J. HZ Univ. 13, 347–351.

[B76] Zhang J. Liu H. Sun J. Li B. Zhu Q. Chen S.. (2012). Arabidopsis fatty acid desaturase FAD2 is required for salt tolerance during seed germination and early seedling growth. PLoS ONE 7:e30355. 10.1371/journal.pone.0030355 22279586 PMC3261201

[B77] Zhang R. Zhang Y. L. Song L. L. Song X. Z. Hänninen H. Wu J. S. (2017). Biochar enhances nut quality of *Torreya grandis* and soil fertility under simulated nitrogen deposition. Forest Ecol. Manage. 391, 321–329. 10.1016/j.foreco.2017.02.036

[B78] Zhang Y. L. Hu Y. Y. Luo H. H. Chow W. S. Zhang W. F. (2011). Two distinct strategies of cotton and soybean differing in leaf movement to perform photosynthesis under drought in the field. Funct. Plant Biol. 38, 567–575. 10.1071/FP11065 32480909

[B79] Zhou Z. Wang M. J. Zhao S. T. Hu J. J. Lu M. Z. (2010). Changes in freezing tolerance in hybrid poplar caused by up- and down-regulation of *PtFAD2* gene expression. Transgenic Res. 19, 1842–1853. 10.1007/s11248-009-9349-x 20012191

