# Supplementary figures and images for: Novel Insights into the Influence of Seed Sarcotesta Photosynthesis on Accumulation of Seed Dry Matter and Oil Content in Torreya grandis cv. “Merrillii”
Source: Front Plant Sci. 2018 Jan 9;8:2179. doi: 10.3389/fpls.2017.02179 (PMC5767305; doi:10.3389/fpls.2017.02179)

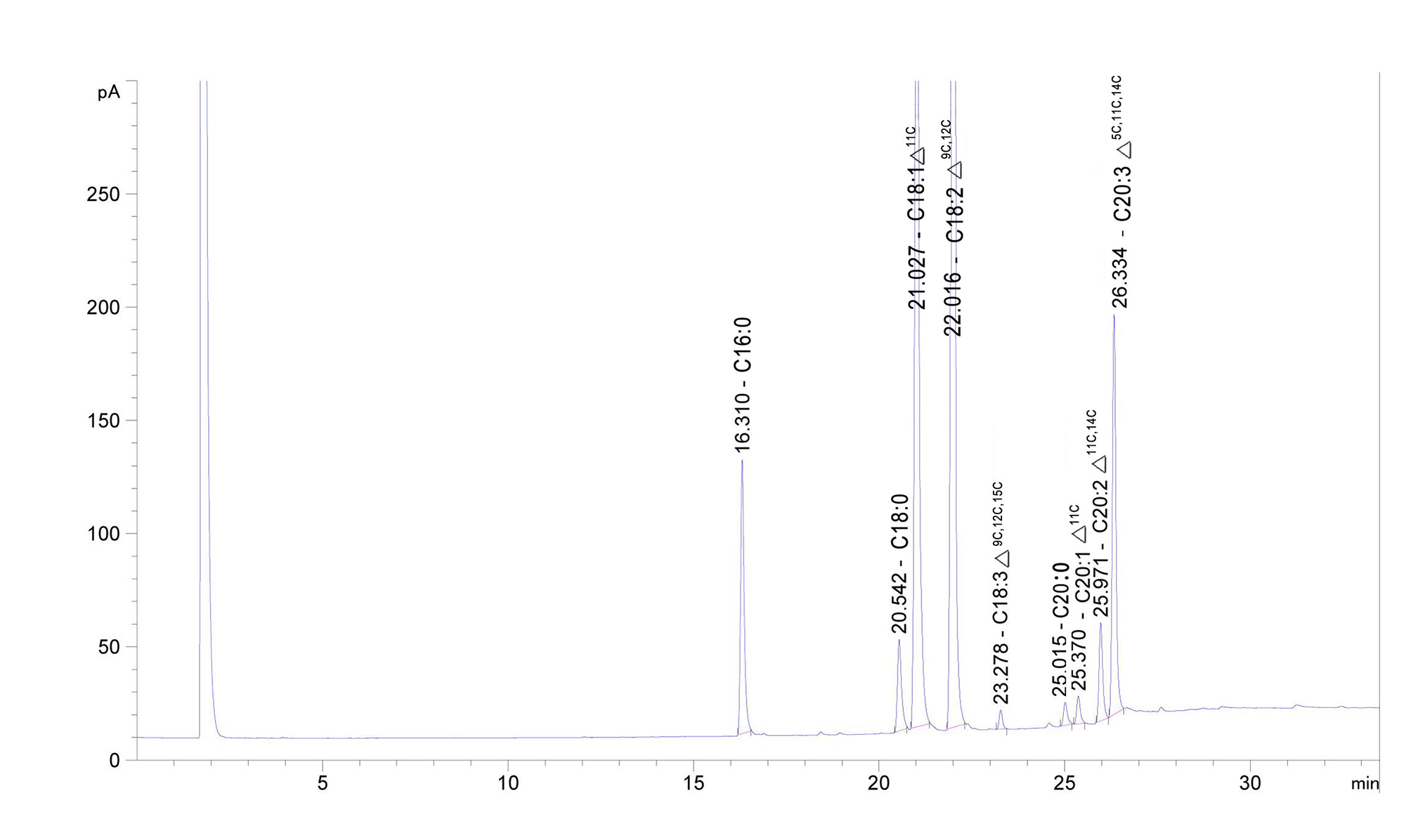

Supplement: Figure S1 — The standard gas chromatogram of the main fatty acids in seeds of Torreya grandis cv. “Merrillii.” [file Image1.tif]

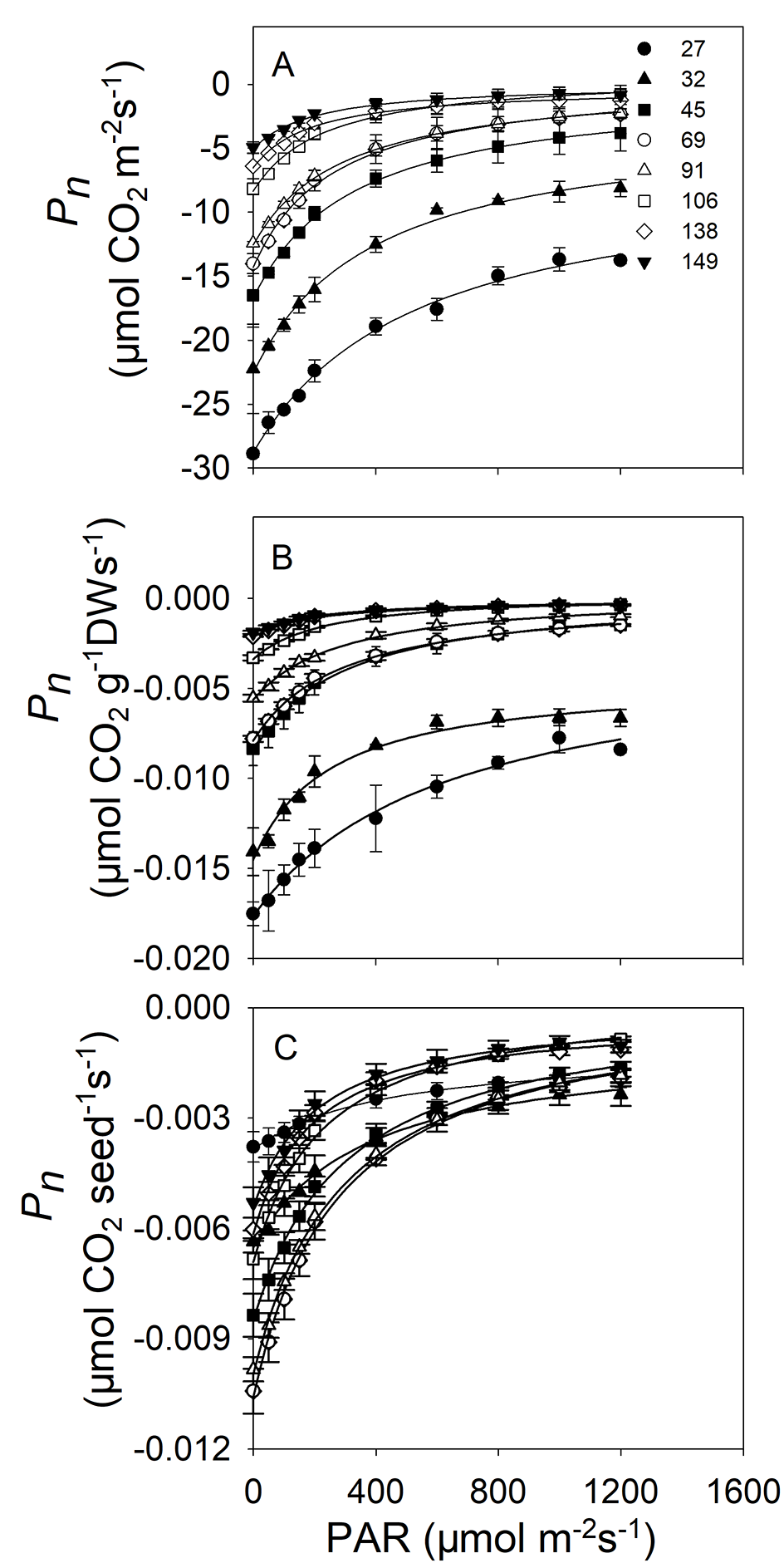

Supplement: Figure S2 — Light responses of net photosynthesis Pn in seeds of Torreya grandis cv. “Merrillii” during seed development; expressed on surface area (A), dry weight (B), and single seed (C) basis. For the Pn values mean ± SD is indicated. Different letters denote significant differences at P ≤ 0.05 level. n = 4. The light responses were measured on eight dates indicated by the symbols and DASP values in the upper right-hand corner of the uppermost panel. DASP, days after seeds protrusion. Note: The values of Pn are negative because of high rates of respiration. The rates were high because the measurements were carried out with intact seed containing also the non-photosynthetic embryo in addition to the photosynthetic sarcotesta. The absolute value of the negative Pn indicates the re-fixation by the sarcotesta photosynthesis of the CO2 released from respiration. See introduction of the main text for further explanation. [file Image2.tif]
