# Supplementary material for: Novel Insights into the Influence of Seed Sarcotesta Photosynthesis on Accumulation of Seed Dry Matter and Oil Content in Torreya grandis cv. “Merrillii”
Source: Front Plant Sci. 2018 Jan 9;8:2179. doi: 10.3389/fpls.2017.02179 (PMC5767305; doi:10.3389/fpls.2017.02179)
Supplement: Table S1 — The inhibitive effect of different concentrations of DCMU on the ETR of seed in comparison with the control. [file Table1.docx]

Table S1 The inhibitive effect of different concentrations of DCMU on the ETR of seed in comparison with the control.

| **Treatment** | **Concentration (μ M)** | ***ETR*（%**） |
| --- | --- | --- |
| **CK** | / | 100% |
| **DCMU** | 75 | 49%±3 |
| **DCMU** | 60 | 63%±1 |
| **DCMU** | 100 | 32%±1 |

Note: In our preliminary experiments, we tried to spray the DCMU on the seed. However, it did not work due to the thick wax coat surrounding the seeds. Data are mean±SD.
